# *Barbeya oleoides* Leaves Extracts: In Vitro Carbohydrate Digestive Enzymes Inhibition and Phytochemical Characterization

**DOI:** 10.3390/molecules26206229

**Published:** 2021-10-15

**Authors:** Alaa A. Khojah, Guillermo F. Padilla-González, Ammar Bader, Monique J. S. Simmonds, Michael Munday, Michael Heinrich

**Affiliations:** 1 UCL School of Pharmacy, University College London, London WC1N 1AX, UK; michael.munday@ucl.ac.uk; 2 Department of Pharmacognosy, Faculty of Pharmacy, Umm Al-Qura University, Makkah 21955, Saudi Arabia; ambader@uqu.edu.sa; 3 Royal Botanic Garden, Kew, Richmond, Surrey TW9 3AB, UK; f.padilla@kew.org (G.F.P.-G.); m.Simmonds@kew.org (M.J.S.S.)

**Keywords:** type 2 diabetes mellitus, *Barbeya oleoides*, α-glucosidase, α-amylase, LC-ESI-MS/MS, aggregation, polyphenols

## Abstract

This study investigated the in vitro inhibitory potential of different solvent extracts of leaves of *Barbeya oleoides* on key enzymes related to type 2 diabetes mellitus (α-glucosidase and α-amylase) in combination with an aggregation assay (using 0.01% Triton X-100 detergent) to assess the specificity of action. The methanol extract was the most active in inhibiting α-glucosidase and α-amylase, with IC_50_ values of 6.67 ± 0.30 and 25.62 ± 4.12 µg/mL, respectively. However, these activities were significantly attenuated in the presence of 0.01% Triton X-100. The chemical analysis of the methanol extract was conducted utilizing a dereplication approach combing LC-ESI-MS/MS and database searching. The chemical analysis detected 27 major peaks in the negative ion mode, and 24 phenolic compounds, predominantly tannins and flavonol glycosides derivatives, were tentatively identified. Our data indicate that the enzyme inhibitory activity was probably due to aggregation-based inhibition, perhaps linked to polyphenols.

## 1. Introduction

Type 2 diabetes mellitus (DM2) is a chronic disease characterized by insulin resistance and improper glucose metabolism, leading to hyperglycaemia. In 2017, it was estimated that over 6% of the global population were affected by DM2 [1]. Uncontrolled hyperglycaemia is associated with severe health complications, including cardiovascular disease, neuropathy, and retinopathy [2]. One of the current therapeutic approaches to treating DM2 is to control postprandial hyperglycaemia by inhibiting carbohydrate digestive enzymes. α-Amylase and α-glucosidase are major digestive enzymes responsible for the breakdown of oligosaccharides and disaccharides into monosaccharides [3]. Acarbose, miglitol, and voglibose are oral α-glucosidase inhibitors commonly prescribed for DM2 patients to delay intestinal absorption of sugar [4]. However, these are associated with undesirable gastrointestinal side effects such as diarrhoea and flatulence [5]. Several reviews described the digestive enzyme inhibitory effects of phytochemicals, including tannin, flavonoids, alkaloids, and terpenoids [6,7,8]. 

Screening of medicinal plants is one strategy to find lead compounds or extracts with potent digestive enzymes inhibiting properties that might counter the undesirable side effects of those in current use [9] and can be a basis for understanding the potential of herbal medicines. Several enzyme-based assays have been used as a starting point for identifying chemical leads. A core limitation is that non-specific effects of extracts or compounds have not been taken into account at an early stage of research [10,11]. For example, polyphenols are known to cause protein aggregation leading to false positives inhibition [12,13,14]. Aggregate-based inhibitors are sensitive to non-ionic detergents, which can disrupt the protein–aggregate interaction and reverse inhibition [15]. Therefore, a detergent-based assay is considered a practical and fast way to identify aggregation-based inhibition [13,14,16].

In this study, we investigated the α-glucosidase and α-amylase inhibitory activity of *Barbeya oleoides* Schweinf. (Barbeyaceae) (*B. oleoides*), a small tree commonly distributed in the mountainous region of Somalia, Ethiopia, Saudi Arabia, and Yemen [17]. *B. oleoides* was selected after a preliminary in vitro screening of α-glucosidase and α-amylase inhibitory potential of seven plants species native to Saudi Arabia (data not shown). *B. oleoides* leaves’ extracts exhibited the highest α-glucosidase and α-amylase inhibitory activities among all tested species. This medicinal plant was traditionally used to manage fever, oedema, infection, and some instances of inflammatory diseases [17].

The first phytochemical work on *B. oleoides* identified barbeyol, an indane-type phenolic compound [17]. In 2012, fifteen compounds were isolated and characterized from aerial parts of *B. oleoides*; these included uvaol, ursolic acid, arjunolic acid, corosolic acid, β-sitosterol-3-*O*-β-d-glucoside, isorhamnetin-4-*O*-glucoside, 3′-methoxyqurecetin-6″-*O*-α-l-rhamnopyranosyl(1–6)β-d-glucopyranoside, d-bornesitol, arjunglucoside I, catechins, and gallocatechins [18]. The later study also reported the antimicrobial activity and spasmolytic activity of *B. oleoides* extracts and a few of these isolated flavonoids and terpenoids. 

The current study focused on the relationship between the chemical profile and enzymatic inhibitory potential (α-glucosidase and α-amylase activities) of *B. oleoides* extracts. Phytochemical profiling was undertaken on the most active extract by conducting a dereplication strategy that utilized ultrahigh-performance liquid chromatography coupled with an electron spray ionization and tandem spectrometry (UPLC-ESI-MS/MS) system. The promiscuous nature and aggregation tendencies were evaluated by conducting a detergent-sensitive enzyme inhibition assay.

## 2. Results and Discussion

### 2.1. In Vitro Enzyme Inhibition

**Figure 1:**
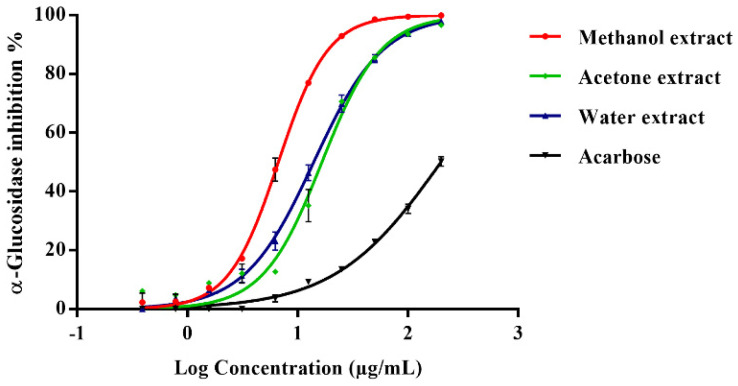
Dose-dependent α-glucosidase inhibition of *B. oleoides* leaves extracts and acarbose (200 μg/mL). Results are shown as mean and SD of three experiments (*n* = 3).

Six extracts of *B. oleoides* (200 μg/mL) of different polarity were assayed (positive control acarbose, 200 μg/mL) (Table 1). In a dose-dependent manner, the acetone, methanol, and water extracts exhibited the most potent α-glucosidase inhibition, with IC_50_ values lower than acarbose (Figure 1). The methanol extract demonstrated potent activity with the lowest IC_50_ value (6.67 ± 0.30 μg/mL) and was the only one to show inhibitory activity against α-amylase (Table 1).The α-amylase inhibition was in a dose-dependent manner and slightly less potent (IC_50_ 25.62 ± 4.12 μg/mL) compared with acarbose (IC_50_ 9.07 ± 0.96 μg/mL) (Figure S45).

**Figure 2:**
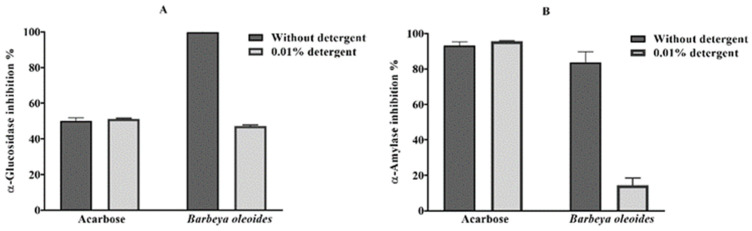
Effect of 0.01% Triton X-100 detergent upon the inhibition of α-glucosidase (**A**) and α-amylase (**B**) by methanol extracts of *B. oleoides* and acarbose (200 µg/mL). The results represent mean and SD of three replicates.

The addition of 0.01% Triton X-100 detergent to α-glucosidase and α-amylase assay reduced the methanolic extract’s (200 μg/mL) inhibition to about half (47.11% ± 0.73) and one-sixth (14.33% ± 4.07), respectively (Figure 2)**.** The addition of the detergent did not affect acarbose inhibitory activities against α-glucosidase and α-amylase (IC_50_ 212.3 ± 7.84 μg/mL and IC_50_ 6.87 ± 0.27 μg/mL, respectively). 

### 2.2. LC-ESI-MS/MS Profiling

**Figure 3:**
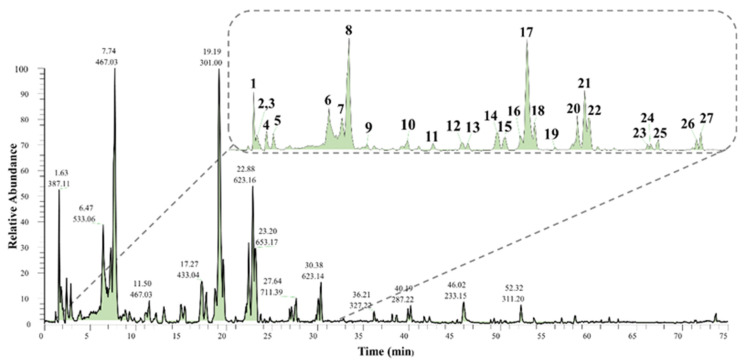
Base peak ion chromatogram in the negative mode of the methanol extract obtained from the leaves of *B. oleoides*.

Since the methanolic extract exhibited the highest inhibitory activity (Table 1), it was subjected to LC-ESI-MS/MS analysis in positive and negative ionization mode simultaneously by switching polarity in the same run. Data generated in the negative mode demonstrated a better ionization efficiency, and therefore, it was selected for the annotation of metabolites (Figure 3). Twenty-seven compounds were successfully separated and detected using LC-ESI-MS/MS. These compounds were tentatively identified through manual inspection of LC-MS data (retention time, accurate mass, elemental composition, and MS^n^ fragments) and by comparison with published data and online databases (Table 2). Twenty-one compounds (**1**–**5**, **7**, **8**, **10**–**19**, **23**, **24**, **26**, and **27**) were identified putatively for the first time in *B. oleoides*, while three compounds (**20**–**22**) had been previously isolated and identified [18]. Interestingly, most of the identified secondary metabolites belong to two main chemical classes: hydrolysable tannins and glycosylated flavonoids (Table 2). However, one disaccharide (**1**) and two unidentified compounds (**9** and **25**) were also detected.

#### 2.2.1. Hydrolysable Tannins and Parent Molecules

**Figure 4:**
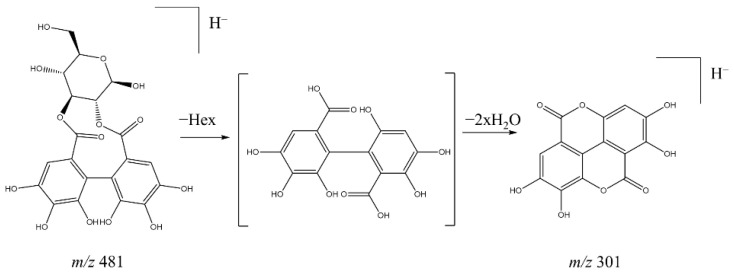
Proposed fragmentation mechanism of HHDP-hexoside to form ellagic acid.

Several hydrolysable tannins, which are characterized by gallic acid and ellagic acid units, were detected in methanol extracts of *B. oleoides* leaves. We identified five structurally related ellagitannins (**2**, **3**, **7**, **8**, and **10**, Figure 3, Table 2) showing a hexahydroxydiphenic acid (HHDP) molecule in their structures (Table 2). Compounds **2** and **3** were identified as two different isomers of HHDP-hexoside by interpretation of their fragmentation pattern and by MS^2^ comparisons with literature data. These compounds showed a deprotonated molecule [M − H]^−^ at 481.0618 and 481.0623 *m*/*z*, respectively. The MS^2^ spectra of both metabolites showed a base peak ion at 301 *m*/*z*, resulting from the neutral loss of a hexose unit (162 Da) and two water molecules to form ellagic acid, following the fragmentation mechanism suggested in Figure 4. The identity of the product ion at 301 *m*/*z* (ellagic acid) was further confirmed by spectral comparisons of its MS^3^ spectra with the data available in the Royal Botanic Garden’s library of MS^2^ data (Figure S4) and published literature [20,21,22]. Given the similarities in the parent ion, UV spectra, and fragmentation pattern to the other ellagitannins, compound **6** likely belongs to the same chemical class as compounds **2** and **3**. This compound was detected as a deprotonated molecule [M − H]^−^ at 533.0562 *m*/*z*, showing an apparent *in*-source dimer at 1067.1179 *m*/*z*. However, detailed analysis of the isotopic pattern showed by the ions at 533 and 1067 *m*/*z* indicated that both ions represent double-charged molecules, suggesting that this compound represents a highly polymerized tannin with a molecular weight above 2000 Da. Therefore, as the molecular weight of this metabolite is outside the employed *m*/*z* range, it was not possible to suggest the identity of compound **6**. Compound **7**, on the other hand, was suggested as a galloyl-HHDP-hexoside based on the interpretation of its mass spectrometry data and literature search [25]. This compound showed a deprotonated molecule [M − H]^−^ at 633.0715 *m*/*z*. The MS^2^ spectra of this metabolite showed a base peak ion at 301 *m*/*z* and a minor fragment at 463 *m*/*z*, likely representing a deprotonated molecule of ellagic acid and ellagic acid linked to a hexose unit, respectively (Table 2). Further analysis of the MS^3^ spectra of the fragment ion at 301 *m*/*z* confirmed its identity as an ellagic acid derivative, given the presence of the diagnostic fragment ions at 257, 229, and 185 *m*/*z* [25]. 

Compounds **8** and **10** showed similar deprotonated molecules [M − H]^−^ at 935.0761 and 935.0757 *m*/*z*, respectively, suggesting their isomeric nature. These compounds were identified as two galloyl-bis-HHDP-hexoside isomers [22,25]. Both compounds exhibited a product ion at 633 *m*/*z* in their MS^2^ spectra, suggesting neutral loss of an ellagic acid molecule (Table 2). However, despite their isomeric nature, these compounds showed different MS^3^ spectra. While compound **10** showed a base peak ion at 301 *m*/*z*, likely representing a deprotonated molecule of ellagic acid, compound **8** was characterized by a base peak ion at 571 *m*/*z*. These differences in retention time and mass spectrometry data could be useful in identifying these isomers in future studies. 

In addition to the ellagitannins described above, two isomers of gallic acid (**4** and **5**) showing distinct MS^2^ spectra were identified (Table 2), as well as three ellagic acid derivatives (**14**, **15**, and **17**, Table 2). Compounds **4** and **5** showed identical deprotonated molecules [M − H]^−^ at 169.0137 *m*/*z*. Both compounds yielded product ions at 151 and 125 *m*/*z*, formed by the neutral loss of a water molecule and a CO unit, respectively (Table 2). However, the stability of these ions differed among isomers. While compound **4** showed a base peak at 151 *m*/*z*, the base peak of compound **5** was the ion at 125 *m*/*z*. A literature search of such fragmentation pattern suggested that these compounds are likely isomers of gallic acid [23,24]. Among ellagic acids, compound **14** gave a deprotonated ion [M − H]^−^ at 433.0401 *m*/*z* that yielded a base peak ion at 301 *m*/*z* [M − H − 132]^−^, indicating the loss of a pentose unit. The MS^3^ spectrum of the 301 *m*/*z* ion was consistent with the characteristic fragment ions of ellagic acid (Table 2), thus suggesting that this compound is likely an ellagic acid pentoside [28]. Compound **15** showed similar MS^2^ and MS^3^ spectra to compound **14** (Table 2), but a different precursor ion at 447.0559 *m*/*z,* suggesting the presence of rhamnose instead of a pentose unit, i.e., an ellagic acid rhamnoside [28]. Compound **17** produced a deprotonated [M − H]^−^ at 300.9983 *m*/*z* with further fragments at 284, 257, 229, and 185 *m*/*z*, identical to those generated by ellagic acid [28,29].

#### 2.2.2. Flavonoid Glycosides

In the methanolic extract of *B. oleoides*, glycosylated flavonols represent the chemical class with the highest diversity of identified metabolites. Thirteen flavonols were tentatively assigned based on mass spectrometry data and literature information. Seven myricetin derivatives were detected and represent the structurally most diverse group of flavonols (**11–13**, **18**, **22**, **24**, and **26**, Table 2), followed by quercetin derivatives (**16**, **20**, **21**, **23**, and **27**) and one kaempferol glycoside (**19**).

Among myricetin derivatives, we found three compounds (**11**, **12**, and **13**) containing a myricetin unit as a core molecule and four compounds with the core being a methoxylated myricetin derivative (**18**, **22**, **24**, and **26**). Compounds **12** and **13** were identified as myricetin-*O*-rutinoside and myricetin-*O*-hexoside, respectively (Table 2). Compound **12** exhibited a deprotonated ion [M − H]^−^ at 625.1390 *m*/*z*, with two major fragment ions at 317 and 316 *m*/*z*, originating from the sequential loss of a rutinoside molecule and a hydrogen radical, respectively. Compound **13** displayed a deprotonated ion [M − H]^−^ at 479.0824 *m*/*z* that further fragmented to produce a base peak ion at 316 *m*/*z*, indicating a loss of a hexose [27]. Since both compounds showed similar MS^3^ spectra with product ions at 287, 271, 179, and 151 *m*/*z*, consistent with those of myricetin [34], these compounds were identified as myricetin glycosides. A similar fragmentation pattern was observed for compound **11**, assigned as myricetin-galloyl-hexoside [27] with a deprotonated molecule [M − H]^−^ at 631.0913 *m*/*z.* The CID fragmentation of the parent molecule produced product ions at 479 *m*/*z* [M–152–H]^−^ and 317 *m*/*z* [M–152–162 –2H]^−^, indicating the losses of a galloyl (152 Da) and hexose units (162 Da). The product ion at 317 *m*/*z* further fragmented to produce ions at 287, 271, 179, and 151 m/z, consistent with the MS^2^ spectra of myricetin [34]. Therefore, compound **11** was suggested as myricetin-galloyl-hexoside [27]. 

Among the four methoxylated derivatives of myricetin, **18** and **24** were identified as two mono-methoxylated derivatives, while **22** and **26** were assigned as dimethoxylated molecules (Table 2). Compound **18** displayed a deprotonated molecule [M − H]^−^ at 639.1545 *m*/*z*, and a base peak ion at 331 *m*/*z*, along with less abundant peak at 316 *m*/*z* in its MS^2^. These fragments suggested the neutral losses of rutinose (308 Da) and CH_3_ group (15 Da), respectively. The MS^3^ spectra of the ion at 316 *m*/*z* showed a base peak at 287, which typifies myricetin along with other fragment ions at 271, 179, and 151 *m*/*z* [34]. Therefore, this compound **18** was suggested as methoxy myricetin-*O*-rutinoside. A similar fragmentation pattern was observed for compound **22** (Table 2); however, the presence of a parent ion [M − H]^−^ at 653.1700 *m*/*z* and two consecutive radical losses of 15 Da suggested a dimethoxylated version of compound **18**. Therefore, compound **22** was putatively annotated as dimethoxy myricetin-*O*-rutinoside, previously described from *B. oleoides* [18]. A similar pattern was observed for compound **24** and compound **26** (Table 2). Compound **24** was tentatively assigned as methoxy myricetin-*O*-caffeoyl-rhamnoside. It showed a deprotonated molecule [M − H]^−^ at 639.1338 *m*/*z* and fragment ions at 493 and 331 *m*/*z,* formed after the neutral losses of a rhamnose and a caffeoyl units, respectively. The MS^3^ spectra of the ion at 331 *m*/*z* showed a base peak at 316 *m*/*z*, indicating the loss of CH_3_ group (15 Da). Compound **26** showed a similar fragmentation pattern as 24 (Table 2). However, two losses of methyl radical (15 Da) were observed in its MS^2^ spectra, suggesting di-methoxylated version of compound **24**. Therefore, compound **26** was tentatively assigned as dimethoxy myricetin-*O*-caffeoyl-rhamnoside. 

Among the quercetin derivatives, we found one quercetin glycoside (**16**), two mono-methoxylated quercetin glycosides (**20** and **21**), and two quercetin derivatives containing a caffeoyl ester in addition to the sugar moiety (**23** and **27**). Compound **16** yielded a deprotonated molecule [M − H]^−^ at 609.1437 *m*/*z* and a major fragment at 301 [M–308–H]^−^, indicating the neutral loss of a rutinoside unit. This product ion further fragmented to produce ions 271, 255, 179, and 151 *m*/*z*, consistent with the MS^2^ spectrum of quercetin [30]. Therefore, this compound was tentatively assigned as quercetin-*O*-rutinoside. A similar fragmentation pattern was found for compound **20** and compound **21**. These compounds produced nearly identical deprotonated ions [M − H]^−^ at 623.1597 and 623.1592 *m*/*z* (error 0.8 ppm), respectively, as well as similar MS^2^ and MS^3^ spectra (Table 2). The presence of fragment ions at 315, 271, and 255 *m*/*z* (Table 2) allowed their structural assignment as isomers of methoxyquercetin-*O*-rutinoside, which was previously identified in *B. oleoides* [18]. Based on the interpretation of their fragmentation patterns, compound **23** and compound **27** were assigned as quercetin-*O*-caffeoyl-rhamnoside and methoxyquercetin-*O*-caffeoyl-rhamnoside, respectively (Table 2). Compound **23** showed a deprotonated molecule [M– H]^−^ at 609.1229 *m*/*z*, and fragment ions at 463 and 301 *m*/*z* (MS^2^) formed after the neutral loss of a rhamnoside and a caffeoyl unit, respectively. The MS^3^ spectra of the ion at 301 *m*/*z* was consistent with that of quercetin [30], thus allowing the tentative assignment of this peak as quercetin-*O*-caffeoyl-rhamnoside. Compound **27**, on the other hand, showed a deprotonated molecule [M − H]^−^ at 623.1386 *m*/*z*. The MS^2^ spectra showed fragment ions at 315 and 300 *m*/*z,* indicating the loss of a methyl group (15 Da). The MS^3^ were found similar to those of compound **23**; thus, compound **27** was assigned as a methoxylated version of compound **23**. 

Lastly, one kaempferol derivative (**19**) was detected. This compound showed a deprotonated molecule [M − H]^−^ at 593.1491 *m*/*z* and fragment ions at 327, 285, 255, and 227 *m*/*z* (Table 2). The presence of the base peak ion at 285 *m*/*z*, indicated the neutral loss of a rutinose unit. The MS^3^ spectra of this ion showed fragmentations similar to those of kaempferol [30]. Therefore, compound **19** was assigned as kaempferol-*O*-rutinoside [30,31,32].

#### 2.2.3. Saccharides

Compound **1** was characterized as sucrose by MS^2^ comparisons with the spectra available in the Royal Botanic Gardens’ library of MS^2^ data (Figure S1). This compound was detected as a formate adduct [M + HCOO]^−^ at 387.1135 *m*/*z*. The MS^2^ spectra of the deprotonated molecule [M − H]^−^ at 341 *m*/*z* resulted in a base peak ion at 179 *m*/*z*, corresponding to the loss of a monosaccharide unit. The MS^3^ spectra of this ion showed a base peak ion at 143, 161, and 131 *m*/*z* (Table 2). The MS^2^ and MS^3^ spectra of this compound were found consistent with those of sucrose, previously reported by Schmidt, et al. [35]. 

#### 2.2.4. Unidentified Compound

Compound **9** showed a peak at *m*/*z* 603.0678 [M − H]^−^ that generated product ions at 458 *m*/*z* in MS^2^ (Table 2). This was further fragmented in MS^3^ to yield product ions at *m*/*z* 573, 436, 427, 413, 382, 343, and 275 *m*/*z*. Compound **25** was presented as formate adduct [M + HCOO]^−^ at 711.3938. The MS^2^ spectra showed product ions at 655 and 503 *m*/*z*. No match was found between the given data and published literature. Therefore, compound **8** and compound **25** could not be identified. 

### 2.3. General Discussion

The enzyme assays indicate that the methanol extracts, obtained from *B. oleoides* leaves, showed prominent inhibitory activity against α-glucosidase and α-amylase. However, based on the aggregation assay, these effects were found non-specific. This makes the development of novel drug leads implausible but points to the need to better understand the chemical profile of the extract, which could be used as herbal medical product. 

The presented phenolic profile of *B. oleoides* methanolic extract, at least in part, explains the strong α-glucosidase and α-amylase inhibitory activity. The majority of the detected compounds were phenolics, specifically flavonols glycosides and hydrolysable tannins. Several studies reported potent α-glucosidase and α-amylase inhibitory activities of phenolic-rich extracts [3,36,37,38] and thus were regarded as a potential for modulating postprandial hyperglycaemia [39]. In previous work, tannins-rich extracts exhibited potent enzymatic inhibition on porcine pancreatic α-amylase [40,41]. Individual tannins, including corilagin (galloyl-HHDP-glucose) [35,42,43], along with ellagic acid [42,44,45] and gallic acid [46,47], were all found to be potent inhibitors of α-glucosidase. Furthermore, isolated flavonols including quercetin, myricetin [48,49], and kaempferol-3-*O*-rutinoside [50] revealed strong α-glucosidase inhibitory activities. 

Polyphenols, including tannins and flavonoids, are known to aggregate with protein in aqueous buffers [12,16,42,51,52]. Despite such evidence that polyphenols form aggregates [15,53], many studies claim their therapeutic effects or drugability without considering their promiscuous potential [10]. Compounds exhibiting such inhibitory activity are known as promiscuous inhibitors [10] and their inhibitory behaviour is usually regarded as non-specific [11]. Therefore, identifying such undesirable hits at an early stage of drug discovery is of significant value before interpreting bioassay data. 

Aggregate-based inhibition characterizes by its sensitivity to non-ionic detergent [51,54]. The percentage of enzyme inhibition by most aggregators reduces by at least 2-fold with the addition of 0.01% Triton X-100 [13,55,56]. Detergent-based assay was found to be a practical way of identifying aggregation-based inhibition [13,56] and was successfully implemented to determine the specificity of enzyme inhibition. Previous work has shown that the addition of 0.01% Triton X-100 resulted in a reduction in the inhibitory activity of quercetin against few unrelated enzymes such as lactate dehydrogenase (LDH) and chymotrypsin [51] as well as β-lactamase [56]. In 2020, Szabo, Hamori, and Gyemant [57] studied the effect of 0.01% of Triton X-100 on α-amylase inhibitory activity by gallotannin, extracted from the gull nut of Aleppo oak (from *Quercus infectoria* G.Olivier). The addition of the non-ion detergent resulted in the inhibitory activity reduction of gallotannin, with a 50-fold increase in the IC_50_ value. 

To our knowledge, this is the first study to identify aggregation-based inhibition of α-glucosidase with the addition of 0.01% Triton-X100. We observed that aggregate formation in α-glucosidase assa could be controlled with the addition of 0.01% Triton X-100 without affecting assay performance. This additional step is recommended when using enzyme-based assays to identify specific bioactivity and eliminate any potential biases [51]. 

In the current work, the enzyme inhibitory activity of the methanol extract of leaves of *B. oleoides* was significantly attenuated with the addition of 0.01% non-ionic detergent. The LC-MS data profiling of this active extract revealed the presence of various polyphenols. Therefore, the inhibitory activity observed for *B. oleoides* methanol extract is likely to be aggregates-based and non-specific inhibition.

## 3. Materials and Methods

### 3.1. Chemical and Reagent 

α-Glucosidase from *Saccharomyces cerevisiae* (EC 2.3.1.20), 4-nitrophenyl α-d-glucopyranoside (pNPG, ≥95%), acarbose ≥ 95%, porcine pancreatic α-amylase (EC 3.2.11), 3,5-dinitrosalicyclic acid (DNS), soluble starch from potato, potassium phosphate monobasic, potassium phosphate dibasic, and Triton X-100 were purchased from Sigma-Aldrich (St. Louis, MO, USA). All solvents were of analytical grade and were purchased from Thermo Fisher Scientific (Waltham, MA, USA).

### 3.2. Plant Material

Leaves of *B. oleoides* were collected from Baljarashi (AL-Baha, Saudi Arabia) in August 2017 and identified by A.B. Samples were air-dried, ground, and stored in the refrigerator at 2–4 °C at the University of Umm al-Qura (Makkah, Saudi Arabia). Samples were transported in airtight containers to University College London (London, UK), where a voucher specimen (No. UQU-UCL-22) was deposited in the school’s herbarium. 

### 3.3. Preparation of Plant Extracts 

Powdered plant material (1 g) was extracted by sonication, utilizing six solvents (10 mL) of increasing polarity (i.e., hexane, chloroform, ethyl acetate, acetone, methanol, and water) in consecutive steps. Plant extracts were shaken vigorously for 2 min and sonicated for 1 h using an ultrasonic water bath. Each extract was centrifuged at 4000 rpm for 40 min, and the supernatant was filtered through Whatman No.4 filter paper (Whatman, Cytiva, Marlborough, MA, USA). Filtered extracts were transferred into glass vials, and solvents were left to evaporate at room temperature. To prepare stocks of test samples, dried extracts were dissolved in DMSO at a 20 mg/mL concentration. Aqueous extracts were freeze-dried and dissolved in sodium phosphate buffer (20 mg/mL).

### 3.4. In Vitro Enzyme Inhibitory Assays

#### 3.4.1. α-Glucosidase Assay

The α-glucosidase assay was performed using a slightly modified version of the method proposed by Yuan et al. [58]. A 50 μL aliquot of the test sample and 100 μL of yeast α-glucosidase (1 U/mL of α-glucosidase dissolved in 0.1 phosphate buffer, pH 6.9) were premixed and incubated in 96-well plates for 10 min at 25 °C. After pre-incubation, 50 uL of 0.1 phosphate buffer (pH 6.9) containing 5 mM p-nitrophenyl-α-d-glucopyranoside (pNPG) as the substrate was added to each well at a timed interval. The reaction mixture then was incubated at 25 °C for 5 min. Before and after the incubation, absorbance was measured at 405 nm using Tecan Infinite M200 Pro microplate reader (Tecan, Mannedorf, Switzerland) and Magellan software. The absorbance was compared with a control containing 50 μL of buffer solution instead of the extract. The reading of blank samples containing buffer in place of the enzyme was subtracted from each well. Acarbose (200 μg/mL) was used as the positive control. The percentage of α-glucosidase inhibition was calculated using the following formula:

(1) $$\%\;\mathrm{Activity}=\frac{\left(\Delta \mathrm{Abs}\;\mathrm{of}\;\mathrm{control}-\Delta \mathrm{Abs}\;\mathrm{sample}\right)}{\Delta \mathrm{Abs}\;\mathrm{of}\;\mathrm{control}\;}\;\times \;100$$

#### 3.4.2. α-Amylase Assay

The α-amylase inhibitory activities of *B. oleoides* extracts were assayed based on a method described by Zaharudin et al. [59]. A 100 μL aliquot of the test sample was mixed with 100 μL of 0.02 M sodium phosphate buffer (pH 6.9 with 0.06 M NaCl) containing porcine pancreatic α-amylase (0.5 U/mL) at 20 °C. The mixture was incubated in a 2 mL Eppendorf tube for 10 min at 25 °C. After pre-incubation, 100 μL of 1% starch solution in 0.02 M sodium phosphate buffer was added to the mixture and incubated for 10 min at 25 °C. To terminate the reaction, 200 μL of 96 mM 3,5-dinitrosalicyclic acid colour reagent was added to the mixture. The mixture in the tube was then heated for 5 min in boiling water and cooled to room temperature. The reaction mixture was diluted with distilled water to a final volume of 2 mL. An amount of 200 μL of each mixture was transferred into 96-well microplates, and the absorbance was measured at 540 nm using a microplate reader and Magellan software. The absorbance was compared with a control containing 100 μL of buffer solution instead of the extract. The reading of blank samples that contained buffer in place of the enzyme was subtracted from each well. Acarbose (200 μg/mL) was used as a positive control. The results are expressed as percentage α-amylase inhibition activity and were calculated according to the Formula (1) above. 

#### 3.4.3. Half-Maximal Inhibitory Concentration (IC_50_) Determination 

The IC_50_ values were calculated using the sample’s percentage of enzyme inhibitory activity tested over a range of concentrations (200 to 1.562 μg/mL). 

#### 3.4.4. Identification of Aggregate-Based Inhibition

*B. oleoides* methanol extracts (200 μg/mL) were tested for enzyme inhibition in the presence of non-ionic detergent. α-Glucosidase and α-amylase inhibitory assays were conducted as described, with the exception that a freshly prepared Triton X-100 was added to the buffer of sample stock solution (0.01% (*v*/*v*) total assay volume). Acarbose was used as negative control (non-aggregator). 

#### 3.4.5. Statistical Analysis 

All experiments were performed in triplicate. Mean and standard deviation (SD) were calculated utilizing Microsoft Excel. The IC_50_ values were estimated by a nonlinear curve that was fitted using GraphPad Prism^®^ program (Version 7.03).

### 3.5. LC-ESI-MS/MS Analysis of B. oleoides Methanol Extract

#### 3.5.1. Sample Preparation 

Methanol extracts of *B. oleoides* were dissolved in methanol (anhydrous solvent—synthesis grade) at concentrations of 50 mg/ mL and filtered through 0.22 μm pore size PTFE membrane. A volume of 1.5 mL of supernatant was transferred to 1.5 mL LC-MS amber vials (Thermo Fisher Scientific^TM^). A volume of 5 μL of the methanol extract was injected into the LC-ESI-MS/MS system. All solvents used were LC-MS grade (Fisher Scientific UK Ltd., Loughborough, UK).

#### 3.5.2. Instrumentation and Analytical Condition

LC-MS was performed utilizing a Thermo Fisher Scientific system (Waltham, MA, USA) equipped with Xcalibur software). The LC consisted of a Vanquish UHPLC system equipped with a photodiode array detector (PDA) offering 100 Hz data rate. Analytes were separated on a Luna C18 column 150 mm × 3 mm, 3 μm (Phenomenex, Torrance, CA, USA) using 400 μL/min eluent gradient of 0:90:10 to 90:0:10 (methanol/water/acetonitrile + 1% formic acid) at 30 °C and over 1 h. UV detection was measured between 210 and 550 nm. The LC system was coupled with a high-resolution MS/MS Orbitrap Fusion Trihybrid mass spectrometer equipped with a heated electrospray (HESI) ionization source. Ionization was performed using a capillary temperature of 350 °C, a capillary voltage of 3.5 Kv (positive ionization mode), and −2.5 Kv (negative ionization modes). Total ion chromatograms (TIC) were acquired between 125 and 1800 *m*/*z*. MS/MS spectra were obtained using a collision energy of 35 eV CID and 60,000 FWHM resolution in the full scan mode. Nitrogen gas was used for drying, nebulization, and fragmentation. Four different scan levels were generated during the experiment, including full scan, MS^2^ of the most abundant peak in full scan, MS^3^ of the most intense peak in MS^2^, and MS^3^ of the second-most intense peak in MS^2^.

#### 3.5.3. Data Analysis 

Chromatograms were processed using Xcalibur™ 4.3 Software (Thermo Fisher Scientific), which allowed the observation of retention times, mass to charge ratios (*m*/*z*), and peaks of >1% intensity, as well as fragmentation patterns of parent peaks (MS^2^ and MS^3^ Spectrum). Peaks of ≥10% intensity were diagnosed in full scan mode (MS1), and fragment ions of ≥5% intensity were reported in MS^2^ and MS^3^. The obtained chromatograms of both negative and positive ionization modes were compared with those obtained by blank samples to exclude any peaks produced by external contaminants. 

#### 3.5.4. Metabolite Identification 

The identification of metabolites was performed by comparisons of accurate mass values (<5 ppm accuracy) and MS^2^ spectral match with information available in the MS^2^ spectral library of Royal Botanic Gardens, Kew. This database, accessible through the NIST MS search 2.0 interface, contains more than 200,000 spectra of synthetic and natural products. Compounds not available in this database were identified by comparing their accurate mass and MS^2^ ions with those reported in published literature. Databases searching in the Dictionary of Natural Products (http://dnp.chemnetbase.com) and SciFinder Scholar (https://scifinder.cas.org) were also used to suggest some structural assignments. 

## Figures and Tables

**Table 1:** The percentage of enzymes inhibition at 200 μg/mL and IC_50_ values of crude extracts of *B. oleoides* leaves. Acarbose was used as a positive control (200 µg/mL). Data are represented as mean ± SD of triplicate experiments.

Extracts (200 µg/mL)	α-Glucosidase Assay		α-Amylase Assay	
	% Inhibition	IC_50_ (µg/mL)	% Inhibition	IC_50_ (µg/mL)
Hexane	11.67 ± 7.81	-	NA	-
Chloroform	50.30 ± 1.64	-	NA	-
Ethyl acetate	50.89 ± 7.34	-	NA	-
Acetone	96.65 ± 0.26	16.61 ± 2.33	NA	-
Methanol	99.73 ± 0.30	6.67 ± 0.30	83.84 ± 5.79	25.62 ± 4.12
Water	98.12 ± 0.46	13.99 ± 1.34	NA	-
Acarbose	50.17 ± 1.58	214.3 ± 6.23	93.22 ± 2.13	9.07 ± 0.96

NA, no activity.

**Table 2:** Detailed mass spectrometry information used for the annotation of metabolites in the methanolic leaves extract of *B. oleoides*.

Com. No	RT (min)	UV Max (nm)	Tentative Identification	Molecular Formula	Observed (*m*/*z*)	Theoretical (*m*/*z*)	Error (ppm)	Fragment Ions-MSn (*m*/*z*)	Source/Reference
**1**	1.63	259	Sucrose	C_12_H_22_O_11_	[M + FA]^−^ 387.1135, [M − H]^−^ 341.1078	[M − H]^−^ 341.1089	−3.22	MS^2^(341): 179 bp, 161, 143, 131, 119, 113, 101 MS^3^(179): 161, 149, 143 bp, 131, 119, 113, 101, 89, 71	[19], Kew library
**2**	1.82	260	HHDP-hexoside (isomer 1)	C_20_H_18_O_14_	[M − H]^−^ 481.0618	[M − H]^−^ 481.0623	−1.04	MS^2^(481): 321, 301 bp, 300, 275 MS^3^(301): 284, 257 bp, 229, 201, 185	[20,21,22]
**3**	2.39	262	HHDP-hexoside (isomer 2)	C_20_H_18_O_14_	[M − H]^−^ 481.0623	[M − H]^−^ 481.0623	0	MS^2^(481): 301 bp, 275 MS^3^(301): 284, 257 bp, 229, 201, 185	[20,21,22]
**4**	2.46	270	Gallic acid (isomer 1)	C_7_H_6_O_5_	[M − H]^−^ 169.0137	[M − H]^−^ 169.0142	−2.96	MS^2^(169): 151 bp, 125 MS^3^(151): 123, 95 bp	[23,24]
**5**	2.9	270	Gallic acid (isomer 2)	C_7_H_6_O_5_	[M − H]^−^ 169.0137	[M − H]^−^ 169.0142	−2.96	MS^2^(169): 125 bp, 151 MS^3^(151): 97, 81 bp	[23,24]
**6**	6.47	272	Unknown tannin	>2000	[M–2H]^2−^ 1067.1188	>2000	-	MS^2^(1067): 377, 885, 933, 977, 1005, 1023 bp MS^3^(1023): 377, 533, 631, 703, 721, 859, 885, 903 bp, 933, 1005.	-
**7**	7.31	272	Galloyl-HHDP-hexoside	C_27_H_22_O_18_	[M − H]^−^ 633.0715	[M − H]^−^ 633.0733	−2.84	MS^2^(633): 463, 301 bp MS^3^(301): 284, 257 bp, 229, 201,185	[25]
**8**	7.74	273	Galloyl-bis-HHDP-hexoside (isomer 1)	C_41_H_28_O_26_	[M − H]^−^ 935.0761	[M − H]^−^ 935.0796	−3.74	MS^2^(935): 917, 873, 783, 659, 633 bp, 615, 589, 571 MS^3^(633): 615, 589, 571 bp, 553, 481, 437, 419, 401, 383, 365, 329, 317, 299, 275	[25,26]
**9**	8.9	271	n.i	-	[M − H]^−^ 603.0678	-	-	MS^2^(603): 458 bp MS^3^(458): 573, 436 bp, 427, 413, 382, 343, 275.	-
**10**	11.5	275	Galloyl-bis-HHDP-hexoside (isomer 2)	C_41_H_28_O_26_	[M − H]^−^ 935.0757	[M − H]^−^ 935.0796	−4.17	MS^2^(935): 917, 783, 633 bp, 615, 301 MS^3^(633): 615, 481, 463, 301 bp	[25,26]
**11**	13.14	266	Myricetin-galloyl-hexoside	C_28_H_24_O_17_	[M − H]^−^ 631.0913	[M − H]^−^ 631.0941	−4.43	MS^2^(631): 479 bp, 317 MS^3^(317): 287, 271, 179, 151	[27]
**12**	15.03	354, 266	Myricetin-*O*-rutinoside	C_27_H_30_O_17_	[M − H]^−^ 625.1390	[M − H]^−^ 625.1410	−3.20	MS^2^(625): 317, 316 bp, 287, 271 MS^3^(316): 287, 271, 179, 151	Fragmentation pattern
**13**	15.37	353, 264	Myricetin-*O*-hexoside	C_21_H_20_O_13_	[M − H]^−^ 479.0824	[M − H]^−^ 479.0831	−1.46	MS^2^(479): 317, 316 bp MS^3^(316): 287 bp, 271, 179, 151	[27]
**14**	17.27	361	Ellagic acid-*O*-pentoside	C_19_H_14_O_12_	[M − H]^−^ 433.0401	[M − H]^−^ 433.0413	−2.77	MS^2^(433): 301 bp, 300 MS^3^(301): 284, 257 bp, 244, 229, 201, 185	[28,29], Kew library
**15**	17.78	361	Ellagic acid-*O*-rhamnoside	C_20_H_16_O_12_	[M − H]^−^ 447.0559	[M − H]^−^ 447.0569	−2.24	MS^2^(447): 301 bp, 300 MS^3^(301): 284, 271, 257 bp, 244, 229, 201, 185	[28,29], Kew library
**16**	18.74	355	Quercetin-*O*-rutinoside	C_27_H_30_O_16_	[M − H]^−^ 609.1437	[M − H]^−^ 609.1461	−3.94	MS^2^(609): 301 bp, 300, 271, 255 MS^3^(301): 271, 255, 179 bp, 151	[30]
**17**	19.19	367	Ellagic acid	C_14_H_6_O_8_	[M − H]^−^ 300.9984	[M − H]^−^ 300.9990	−1.99	MS^2^(301): 284, 257 bp, 244, 229, 201, 185	[28,29], Kew library
**18**	19.66	359	Methoxymyricetin-*O*-rutinoside	C_28_H_32_O_17_	[M − H]^−^ 639.1545	[M − H]^−^ 639.1567	−3.44	MS^2^(639): 607, 331 bp, 316, 315, 287, 271 MS^3^(316): 287 bp, 271, 259, 243, 178, 151	Fragmentation pattern
**19**	20.96	348	Kaempferol-*O*-rutinoside	C_27_H_30_O_15_	[M − H]^−^ 593.1491	[M − H]^−^ 593.1512	−3.54	MS^2^(593): 327, 285 bp, 255, 227 MS^3^(285): 267, 257, 255 bp, 241, 229, 227, 213, 197, 151	[30,31,32]
**20**	22.48	355	Methoxyquercetin-*O*-rutinoside (isomer 1)	C_28_H_32_O_16_	[M − H]^−^ 623.1597	[M − H]^−^ 623.1618	−3.37	MS^2^(623): 357, 315 bp, 300, 271 MS^3^(315): 300 bp, 299 MS^3^(300): 271 bp, 255	[18,30,33]
**21**	22.88	355	Methoxyquercetin-*O*-rutinoside (isomer 2)	C_28_H_32_O_16_	[M − H]^−^ 623.1592	[M − H]^−^ 623.1618	−4.17	MS^2^(623): 357, 315 bp, 300, 271 MS^3^(315): 300 bp, 299 MS^3^(300): 271 bp, 255	[18,30,33]
**22**	23.20	359	Dimethoxymyricetin-*O*-rutinoside	C_29_H_34_O_17_	[M − H]^−^ 653.1700	[M − H]^−^ 653.1723	−3.52	MS^2^(653): 345 bp, 330, 315, 287 MS^3^(345): 330 bp, 315 MS^3^(315): 287 bp, 271, 259, 243	[18]
**23**	26.92	311, 265	Quercetin-*O*-caffeoyl-rhamnoside	C_30_H_26_O_14_	[M − H]^−^ 609.1229	[M − H]^−^ 609.1250	−3.45	MS^2^(609): 463 bp, 301 MS^3^(301): 271 bp, 255, 179, 151	Fragmentation Pattern
**24**	27.11	311, 267	Methoxymyricetin-*O*-caffeoyl-rhamnoside	C_31_H_28_O_15_	[M − H]^−^ 639.1338	[M − H]^−^ 639.1355	−2.66	MS^2^(639): 493 bp, 331 MS^3^(331): 316 bp, 315, 179, 151	Fragmentation Pattern
**25**	27.64	281, 265	n.i.	-	[M + FA]^−^ 711.3938, [M − H]^−^ 665.3620	-	-	MS^2^(711): 665, 503 bp MS^3^(503): 505, 485, 453, 441 bp, 421, 409, 403	-
**26**	30.08	311, 266	Dimethoxymyricetin-*O*-caffeoyl-rhamnoside	C_32_H_30_O_15_	[M − H]^−^ 653.1491	[M − H]^−^ 653.1512	−3.32	MS^2^(653): 345 bp, 330, 315, 287 MS^3^(345): 330 bp, 315 MS^3^(315): 287 bp, 271, 259, 243	Fragmentation Pattern
**27**	30.38	315, 265	Methoxyquercetin-*O*-caffeoyl-rhamnoside	C_31_H_28_O_14_	[M − H]^−^ 623.1386	[M − H]^−^ 623.1406	−3.21	MS^2^(623): 315 bp, 300 MS^3^(300): 271 bp, 255, 227, 151	Fragmentation Pattern

RT, retention time; FA, formic acid; bp, base peak.

## Data Availability

The authors confirm that most of the data supporting the findings of this study are available within the article and its Supplementary Materials. Raw data are available from the corresponding author (A.A.K.) on request.

## Supplementary Materials

The following are available online.

## Sample Availability

Tested plant sample are available from the corresponding author (A.A.K.) on request.
