# Supplementary material for: Barbeya oleoides Leaves Extracts: In Vitro Carbohydrate Digestive Enzymes Inhibition and Phytochemical Characterization
Source: Molecules. 2021 Oct 15;26(20):6229. doi: 10.3390/molecules26206229 (PMC8540058; doi:10.3390/molecules26206229)

Supporting Information

Barbeya oleoides leaves extracts: *In vitro* carbohydrate digestive enzymes inhibition and phytochemical characterization

Alaa A. Khojah [1,3,*], Guillermo F. Padilla-González [2], Ammar Bader [3], Monique J. S. Simmonds [2], Michael Munday [1], Michael Heinrich [1,*]

[1] UCL School of Pharmacy, University College London, London WC1N 1AX, UK; michael.munday@ucl.ac.uk (M.M.)

[2] Royal Botanic Garden, Kew, Richmond, Surrey TW9 3AB, UK; m.Simmonds@kew.org (M.S.J.S.); f.padilla@kew.org (G.F.P.)

[3] Department of Pharmacognosy, Faculty of Pharmacy, Umm Al-Qura University, Makkah 21955, Saudi Arabia; ambader@uqu.edu.sa (A.B.)

* Correspondence: a.khojah@ucl.ac.uk (A.K.); m.heinrich@ucl.ac.uk (M.H.)

List of Supplementary Material

**No.**	**Content**	Page
**Figure S1**	**MS^2 Spectral matching of compound 1 with Kew Library**	**1**
**Figure S2**	**MS^2 spectrum of compound 2**	**2**
**Figure S3**	**MS^2 spectrum of compound 3**	**3**
**Figure S4**	**MS^3 Spectral matching of compound 3 with Kew Library**	**4**
**Figure S5**	**Proposed fragmentation pattern of compounds 2 and 3**	**5**
**Figure S6**	**MS^2 spectrum of compound 6**	**6**
**Figure S7**	**MS^2 spectrum of compound 5**	**7**
**Figure S8**	**MS^2 spectrum of compound 6**	**8**
**Figure S9**	**MS^2 spectrum of compound 7**	**9**
**Figure S10**	**MS^2 spectrum of compound 8**	**10**
**Figure S11**	**MS^2 spectrum of compound 9**	**11**
**Figure S12**	**MS^3 spectrum of compound 9**	**12**
**Figure S13**	**MS^2 spectrum of compound 10**	**13**
**Figure S14**	**MS^3 spectrum of compound 10**	**14**
**Figure S15**	**MS^2 spectrum of compound 11**	**15**
**Figure S16**	**MS^3 spectrum of compound 11**	**16**
**Figure S17**	**MS^2 spectrum of compound 12**	**17**
**Figure S18**	**MS^3 spectrum of compound 12**	**18**
**Figure S19**	**MS^2 spectrum of compound 13**	**19**
**Figure S20**	**MS^3 spectrum of compound 13**	**20**
**Figure S21**	**MS^2 spectrum of compound 14**	**21**
**Figure S22**	**MS^3 spectrum of compoud 14**	**22**
**Figure S23**	**MS^2 spectrum of compound 15**	**23**

**Figure S24**	**MS^3 spectrum of compound 15**	**24**
**Figure S25**	**MS^2 spectrum of compound 16**	**25**
**Figure S26**	**MS^3spectrum of compound 16**	**26**
**Figure S27**	**MS^2 spectrum of compound 17**	**27**
**Figure S28**	**MS^2 spectrum of compound 18**	**28**
**Figure S29**	**MS^3 spectrum of compound 18**	**29**
**Figure S30**	**MS^2 spectrum of compound 19**	**30**
**Figure S31**	**MS^2 spectrum of compound 20**	**31**
**Figure S32**	**MS^2 spectrum of compound 21**	**32**
**Figure S33**	**MS^2 spectrum of compound 22**	**33**
**Figure S34**	**MS^3 spectrum of compound 22**	**34**
**Figure S35**	**MS^2 spectrum of compound 23**	**35**
**Figure S36**	**MS^3 spectrum of compound 23**	**36**
**Figure S37**	**MS^2 spectrum of compound 24**	**37**
**Figure S38**	**MS^3 spectrum of compound 24**	**38**
**Figure S39**	**MS^2 spectrum of compound 25**	**39**
**Figure S40**	**MS^3 spectrum of compound 25**	**40**
**Figure S41**	**MS^2 spectrum of compound 26**	**41**
**Figure S42**	**MS^3 spectrum of compound 26**	**42**
**Figure S43**	**MS^2 spectrum of compound 27**	**43**
**Figure S44**	**MS^3 spectrum of compound 27**	**44**
**Figure S45**	**Dose-response curve of α-amylase inhibition by acarbose and methanol extracts obtained from *Barbeya oleoides* leaves (200 µg/ml).**	**45**

Figure S1. MS[2] Spectral matching of compound 1 with Kew Library.

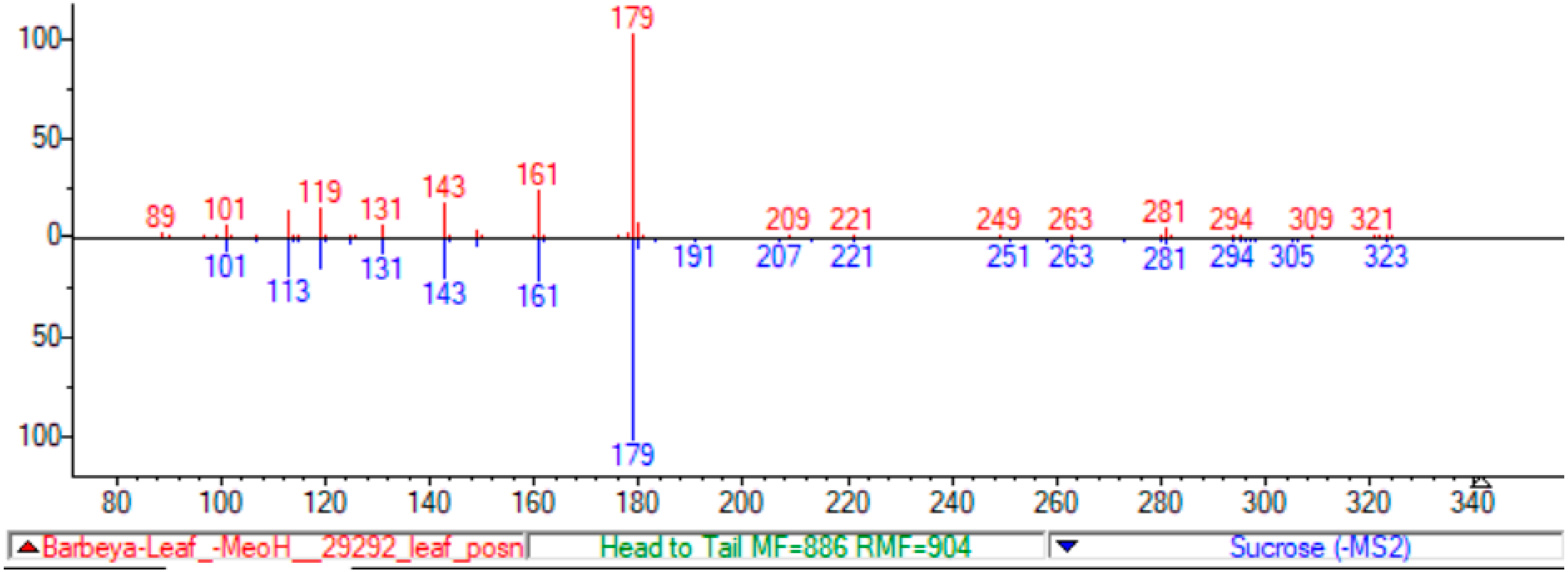

Figure S2. MS^2 spectrum of compound 2.

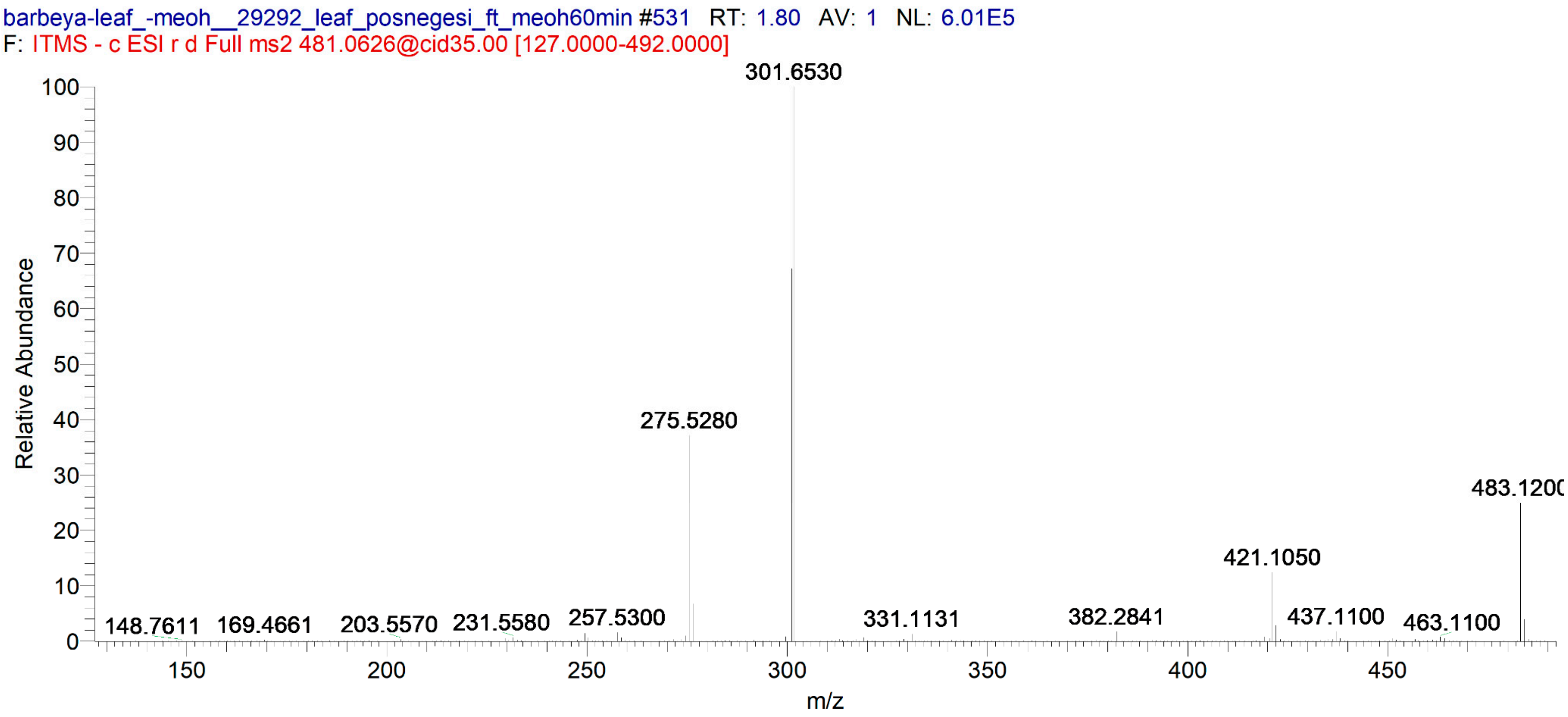

Figure S3. MS^2 spectrum of compound 3.

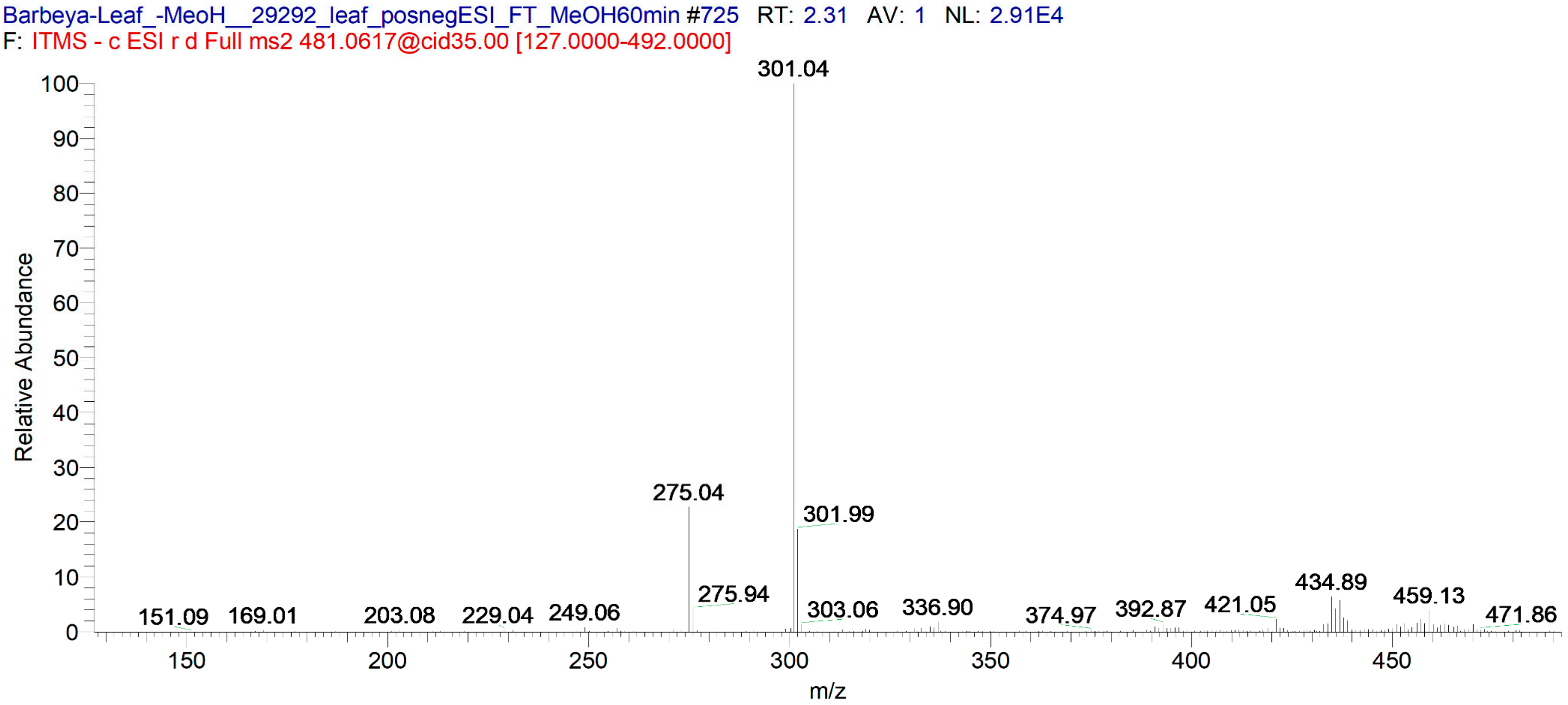

Figure S4. MS^3 Spectral matching of compound 3 with MS^2 Kew Library of ellagic acid.

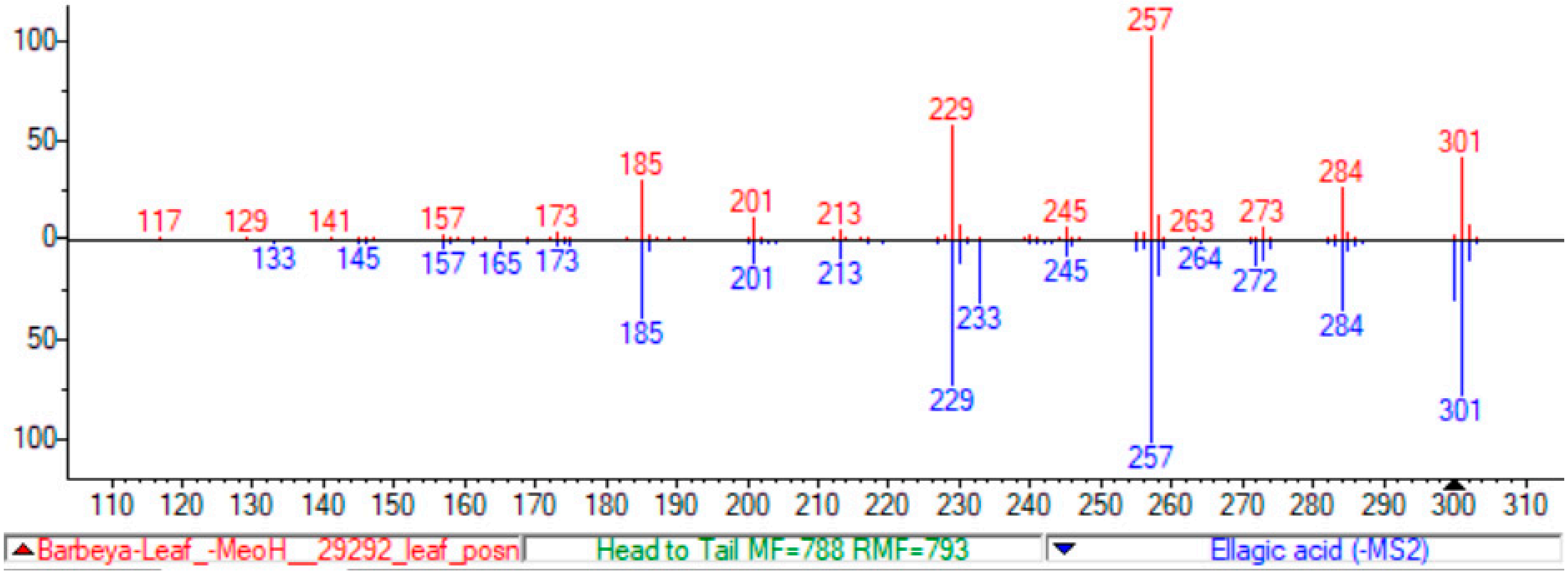

Figure S5. Proposed Fragmentation pattern of compounds 2 and 3.

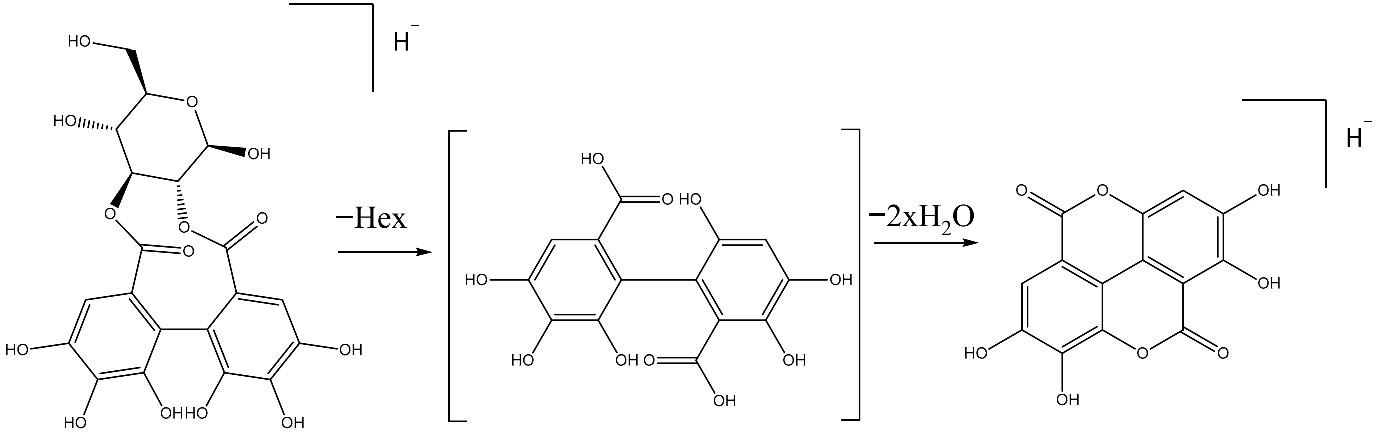

m/z 481

m/z 301

Figure S6. MS^2 spectrum of compound 4.

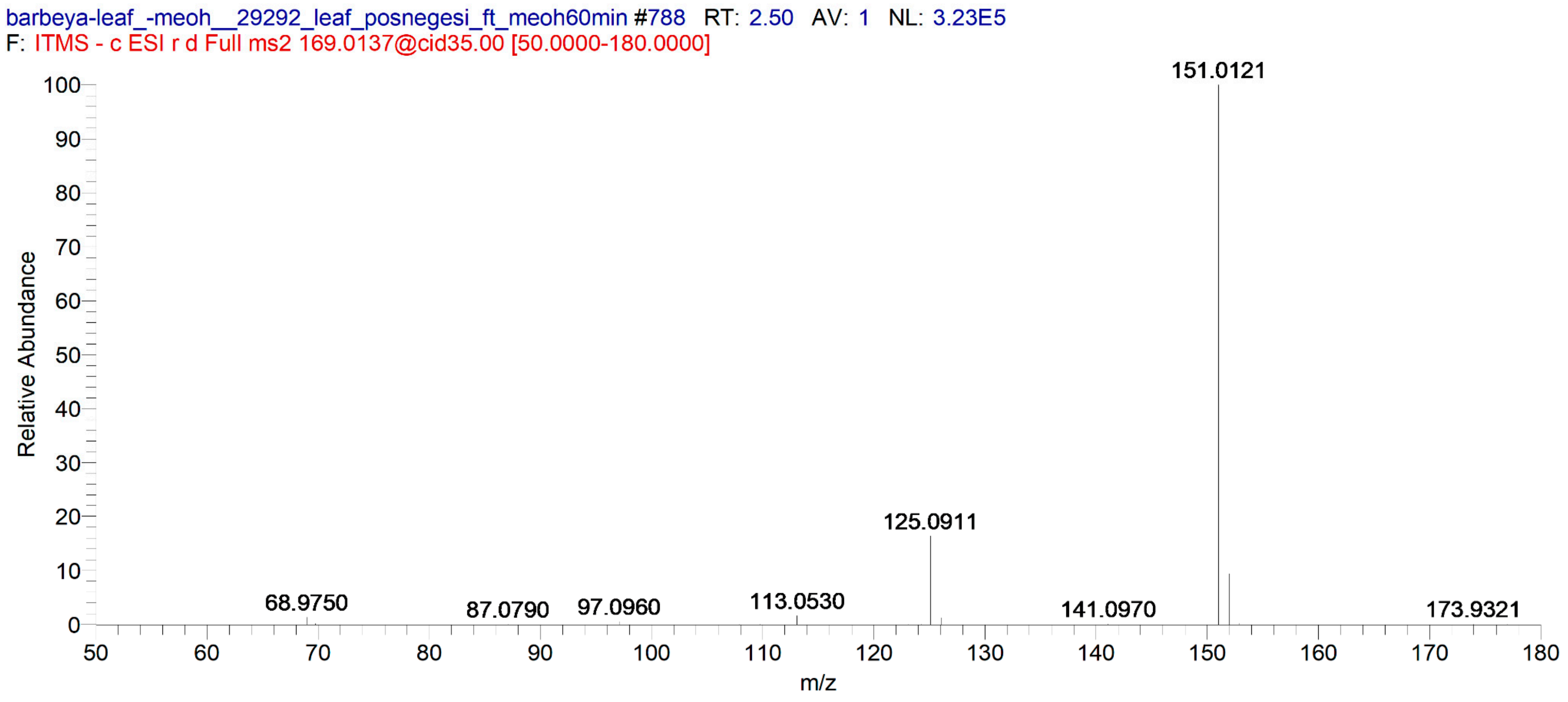

Figure S7. MS^2 spectrum of compound 5.

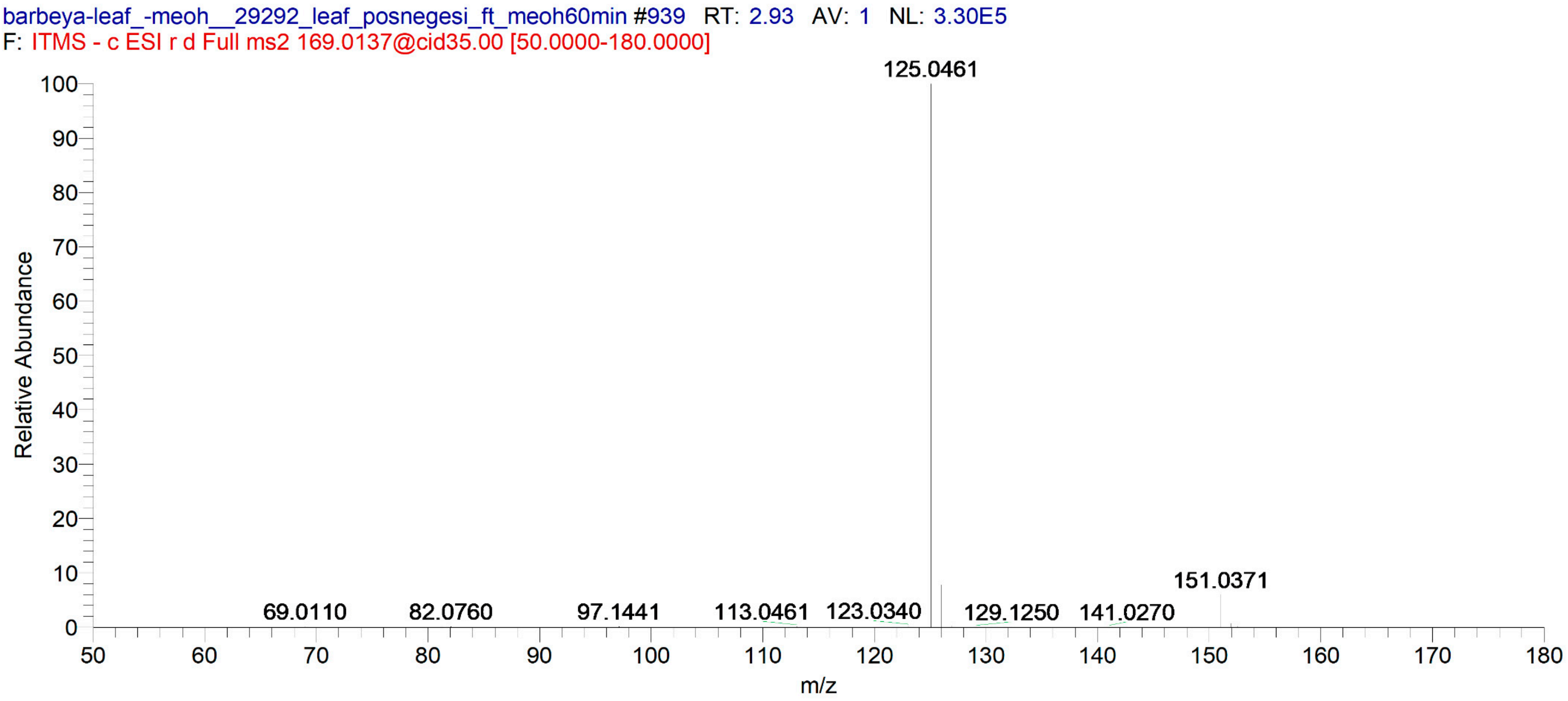

Figure S8. MS² spectrum of compound 6.

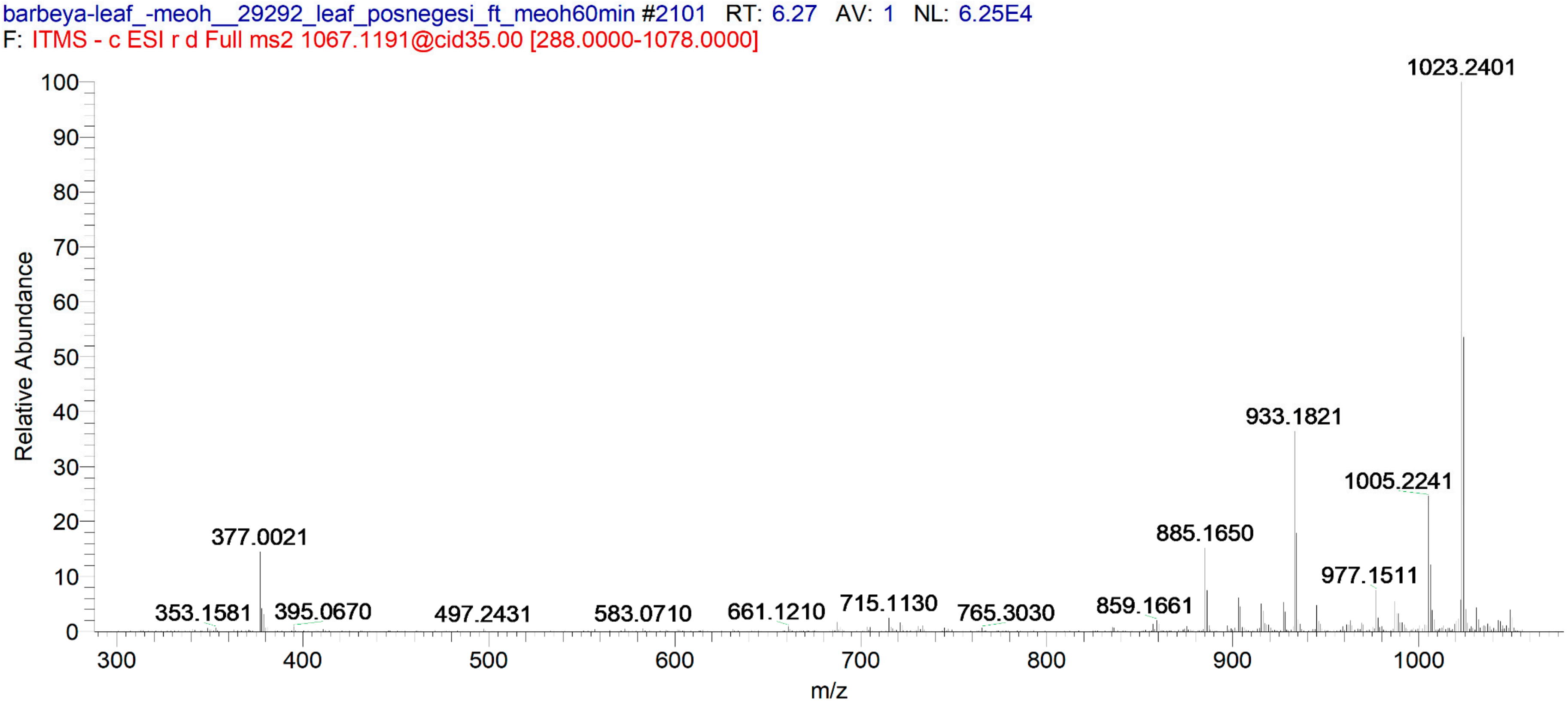

Figure S9. MS^2 spectrum of compound 7.

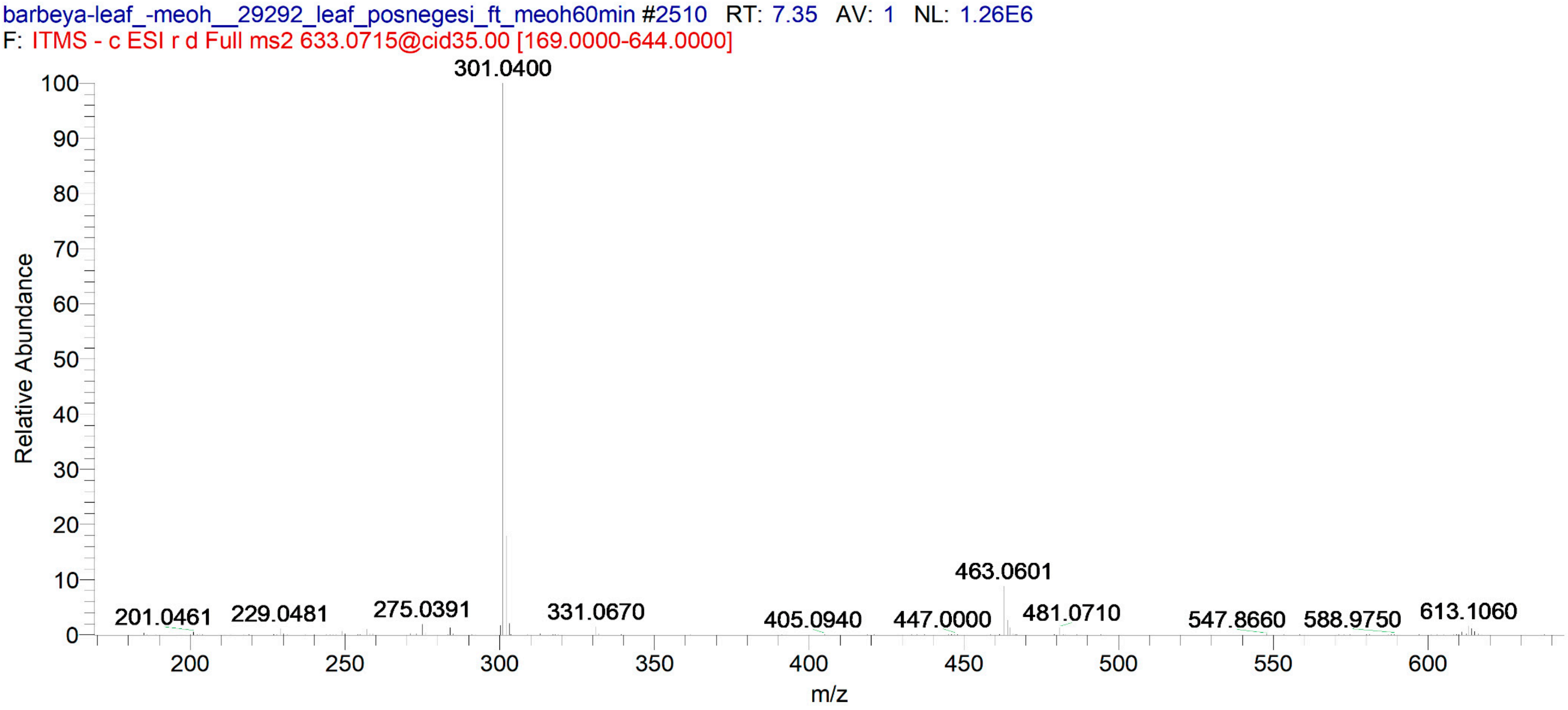

Figure S10. MS^2 spectrum of compound 8.

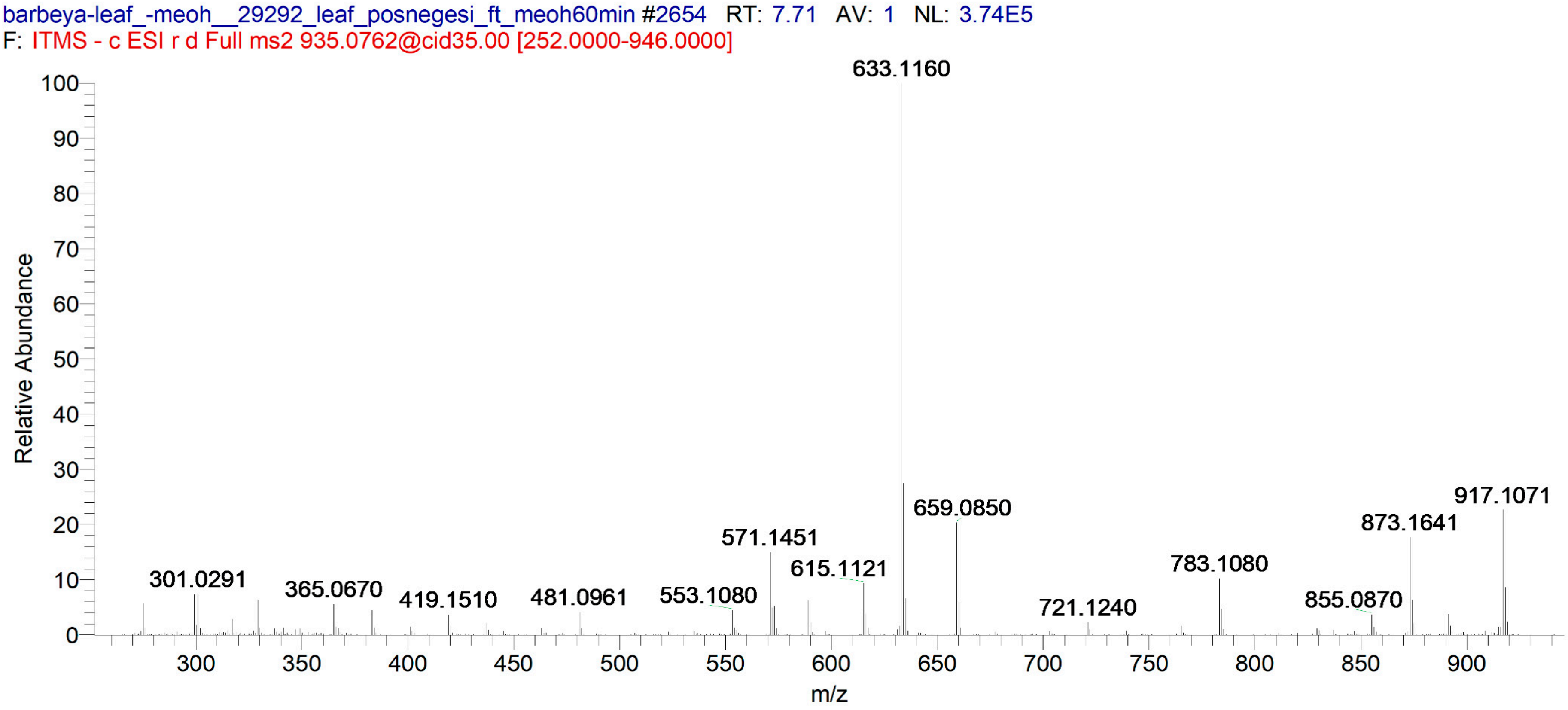

Figure S11. MS^2 spectrum of compound 9.

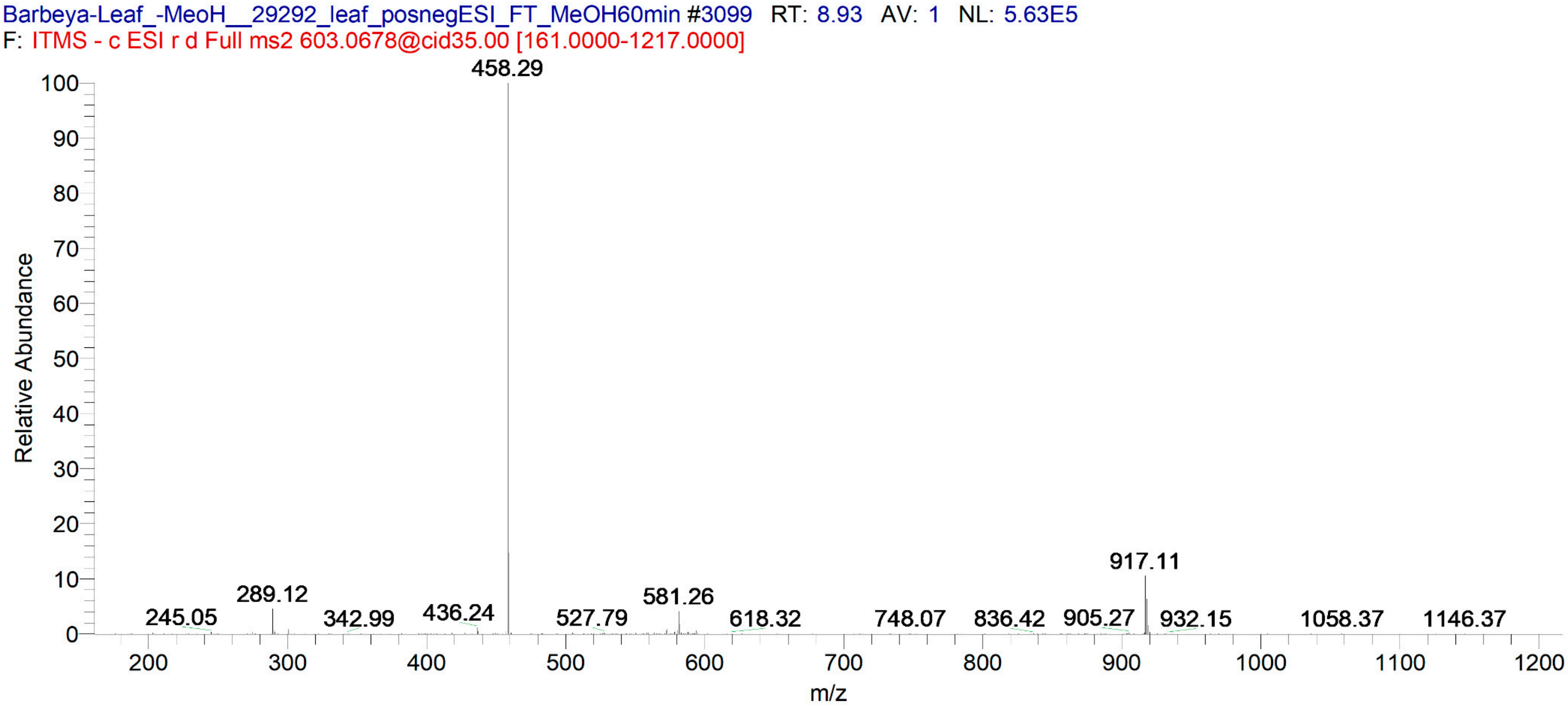

Figure S12. MS^3 spectrum of compound 9.

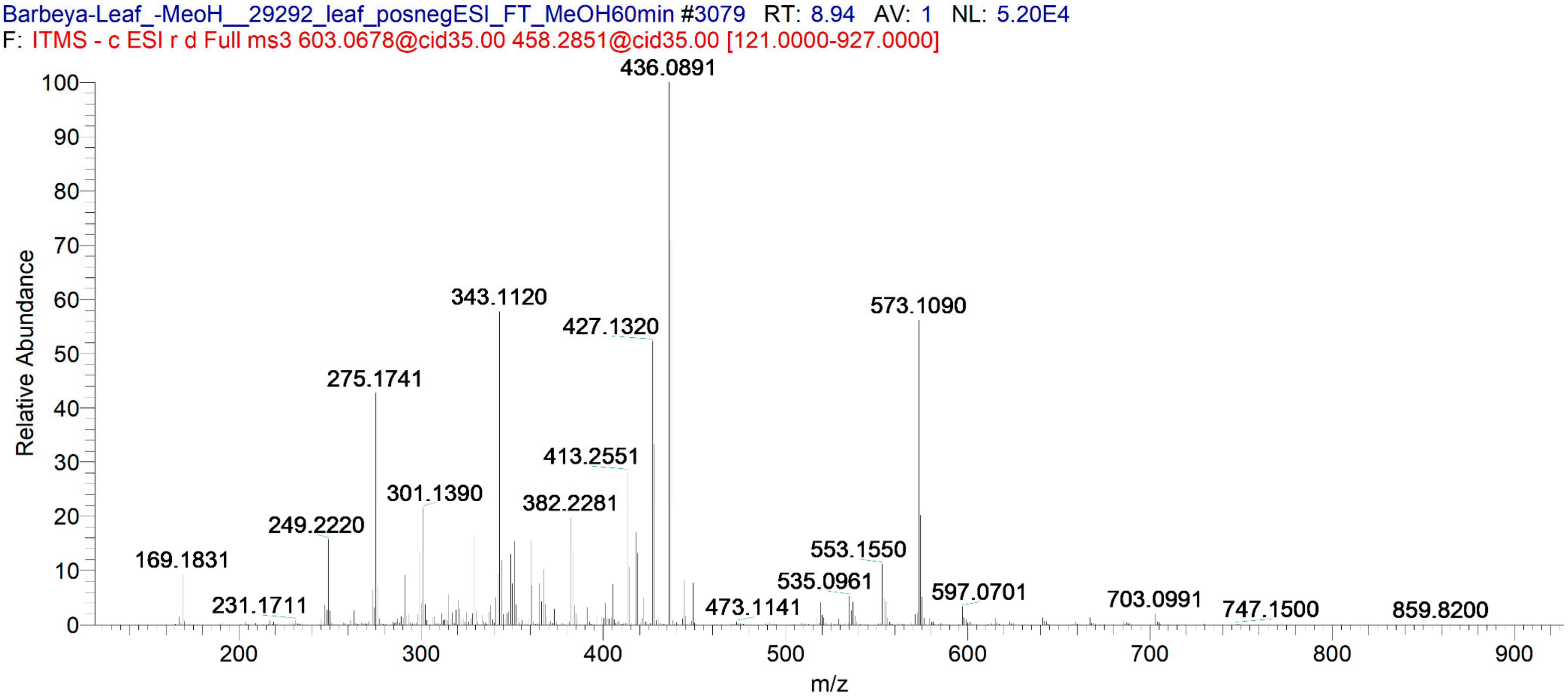

Figure S13. MS^2 spectrum of compound 10.

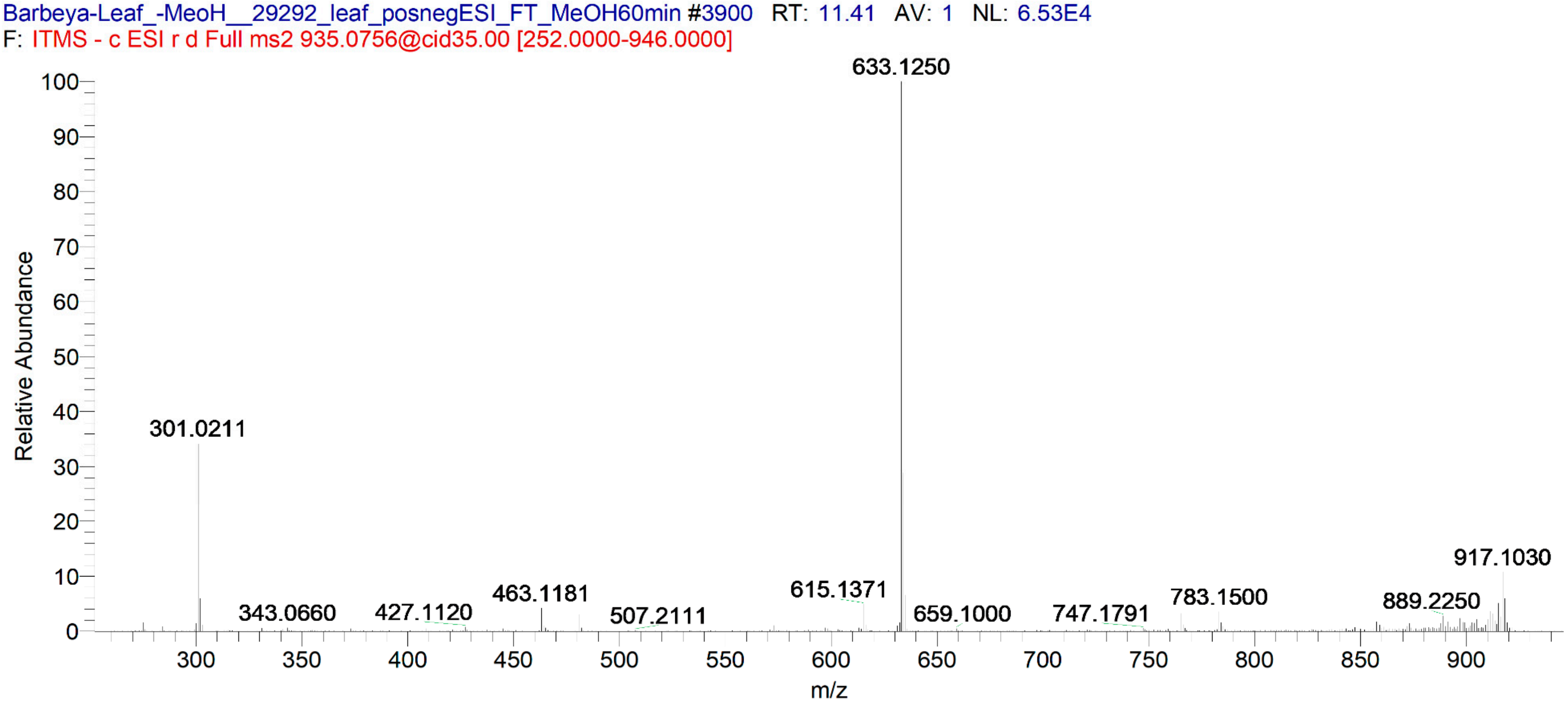

Figure S14. MS^3 spectrum of compound 10

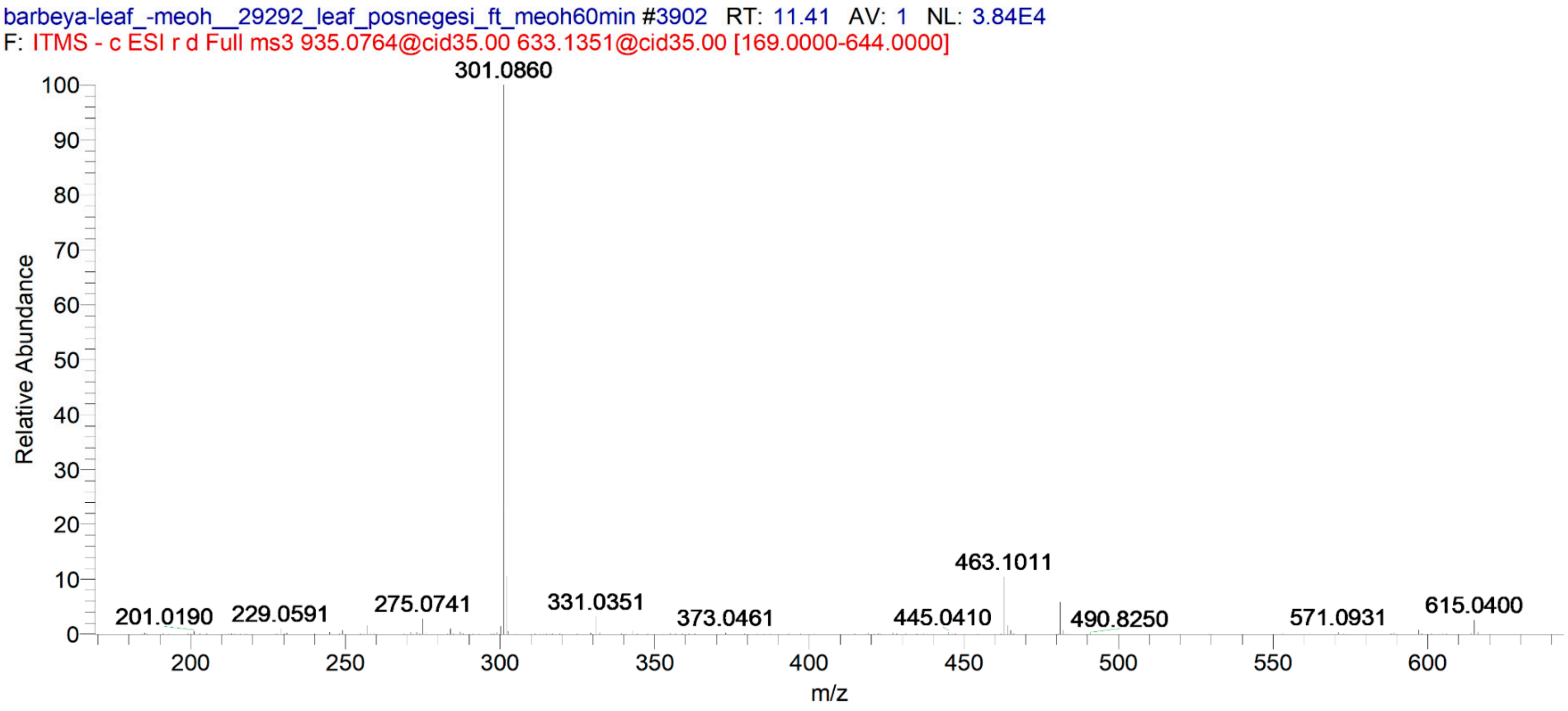

Figure S15. MS^2 spectrum of compound 11.

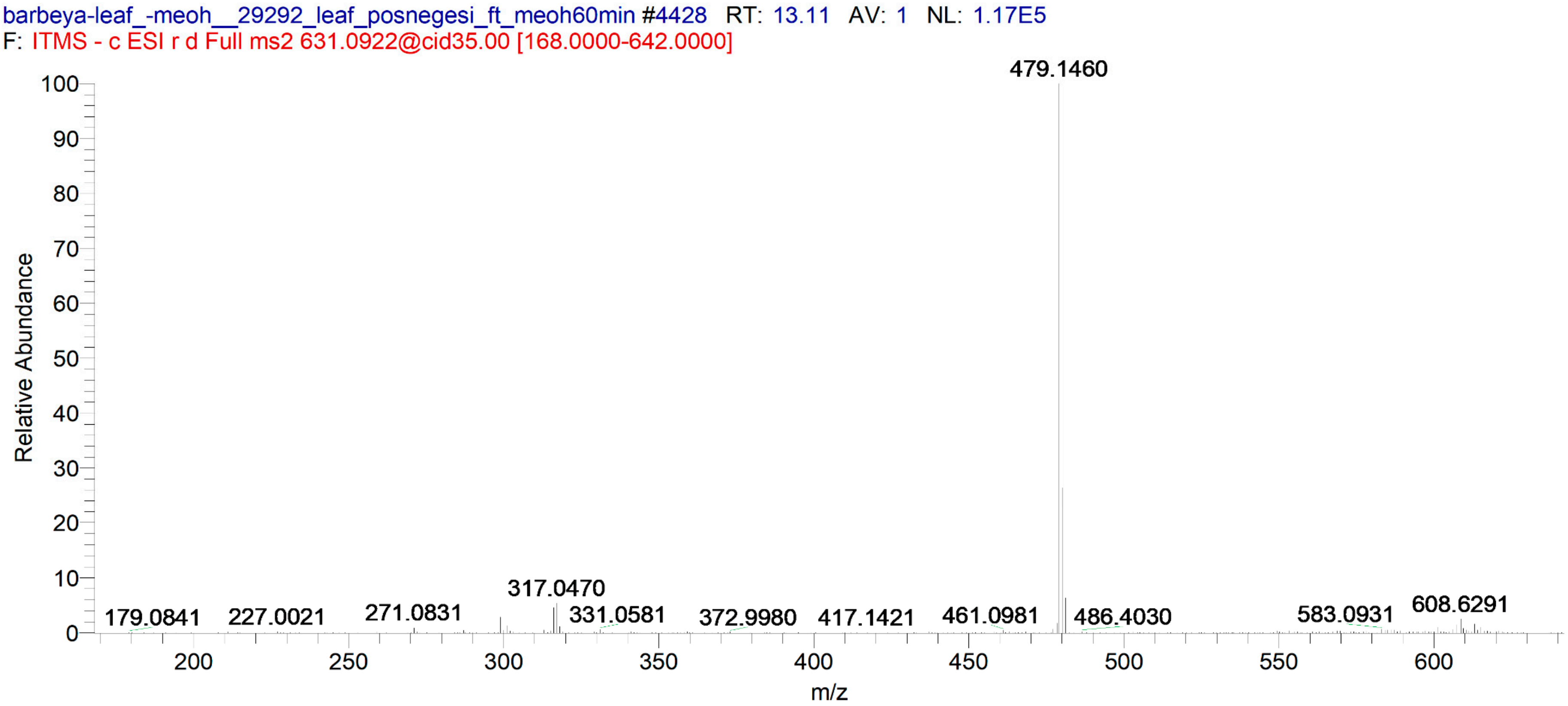

Figure S16. MS^3 spectrum of compound 11.

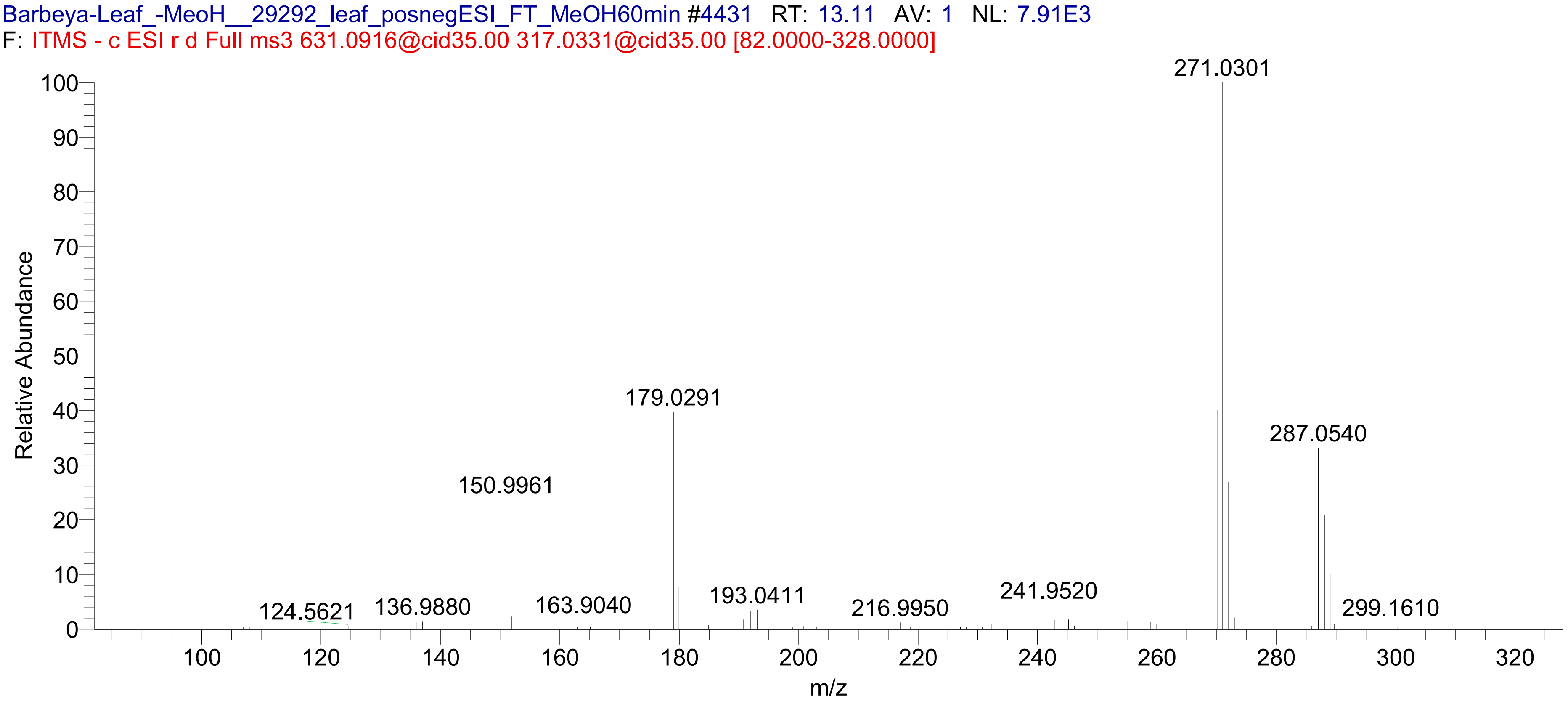

Figure S17. MS^2 spectrum of compound 12.

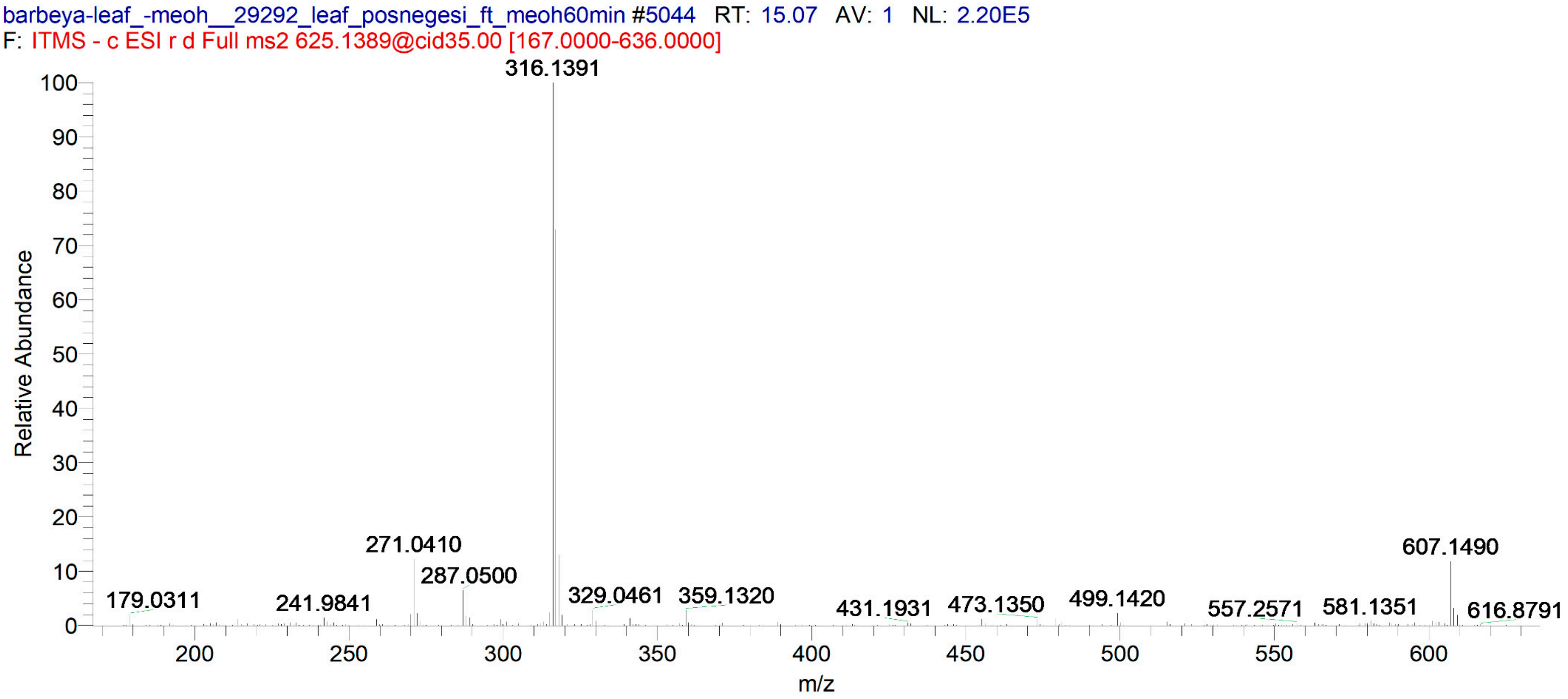

Figure S18. MS^3 spectrum of compound 12.

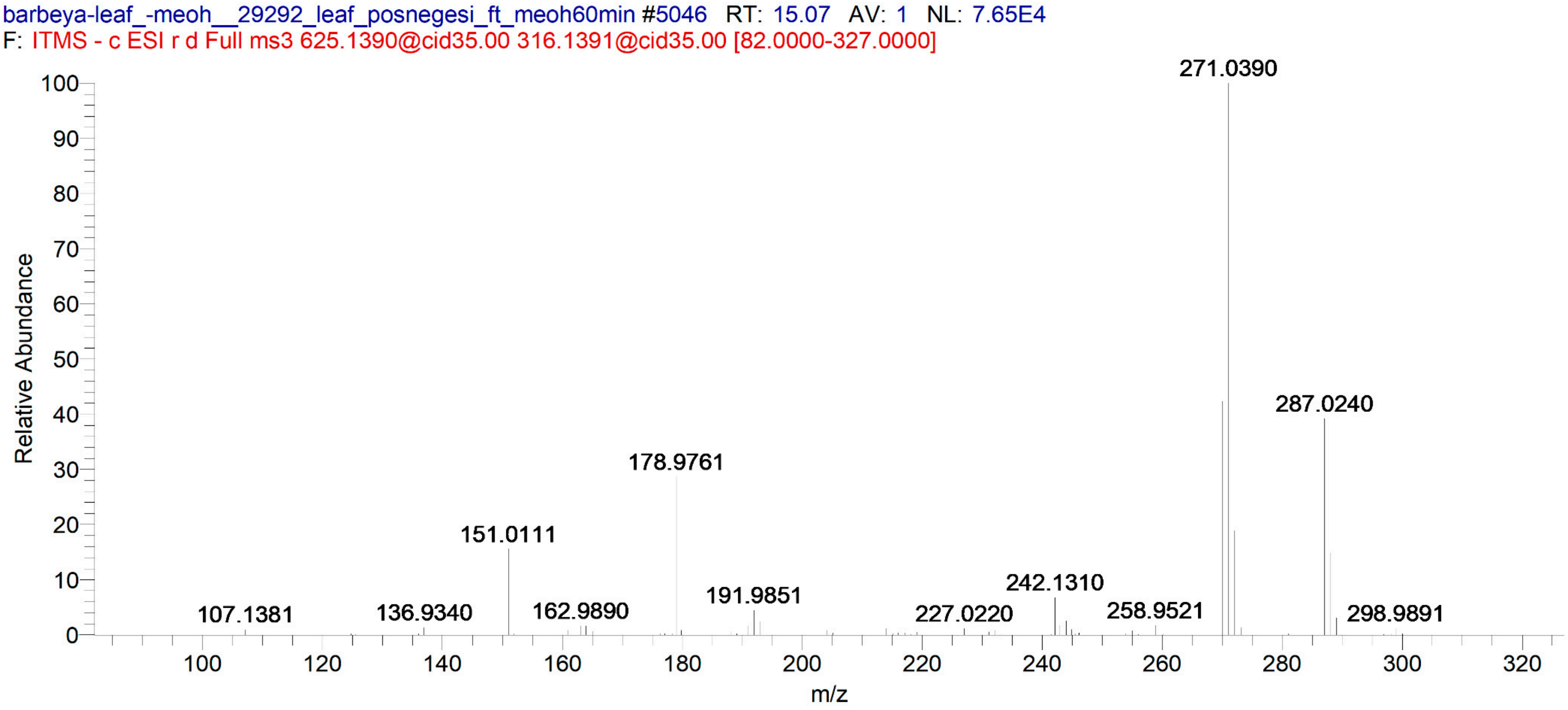

Figure S19. MS^2 spectrum of compound 13.

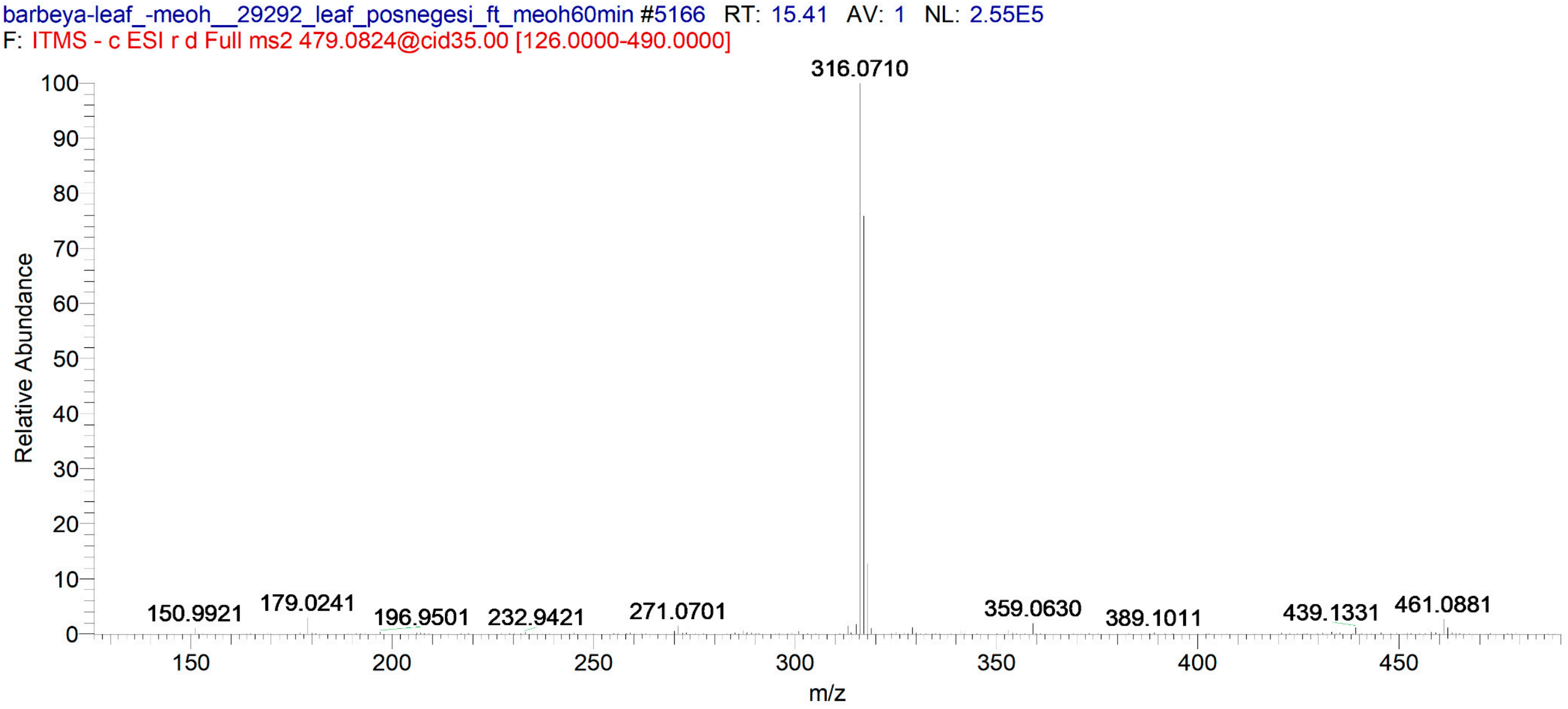

Figure S20. MS^3 spectrum of compound 13.

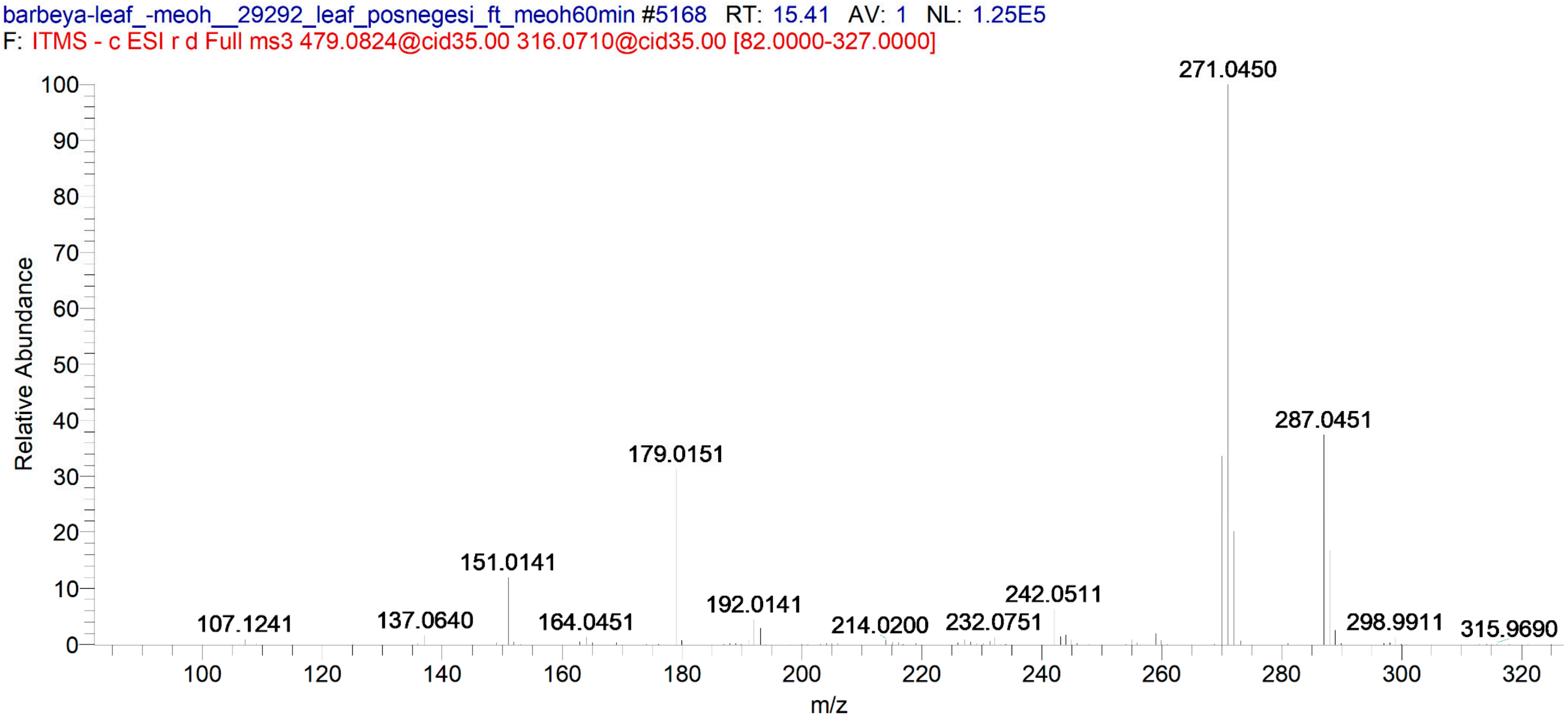

Figure S21. MS^2 spectrum of compound 14.

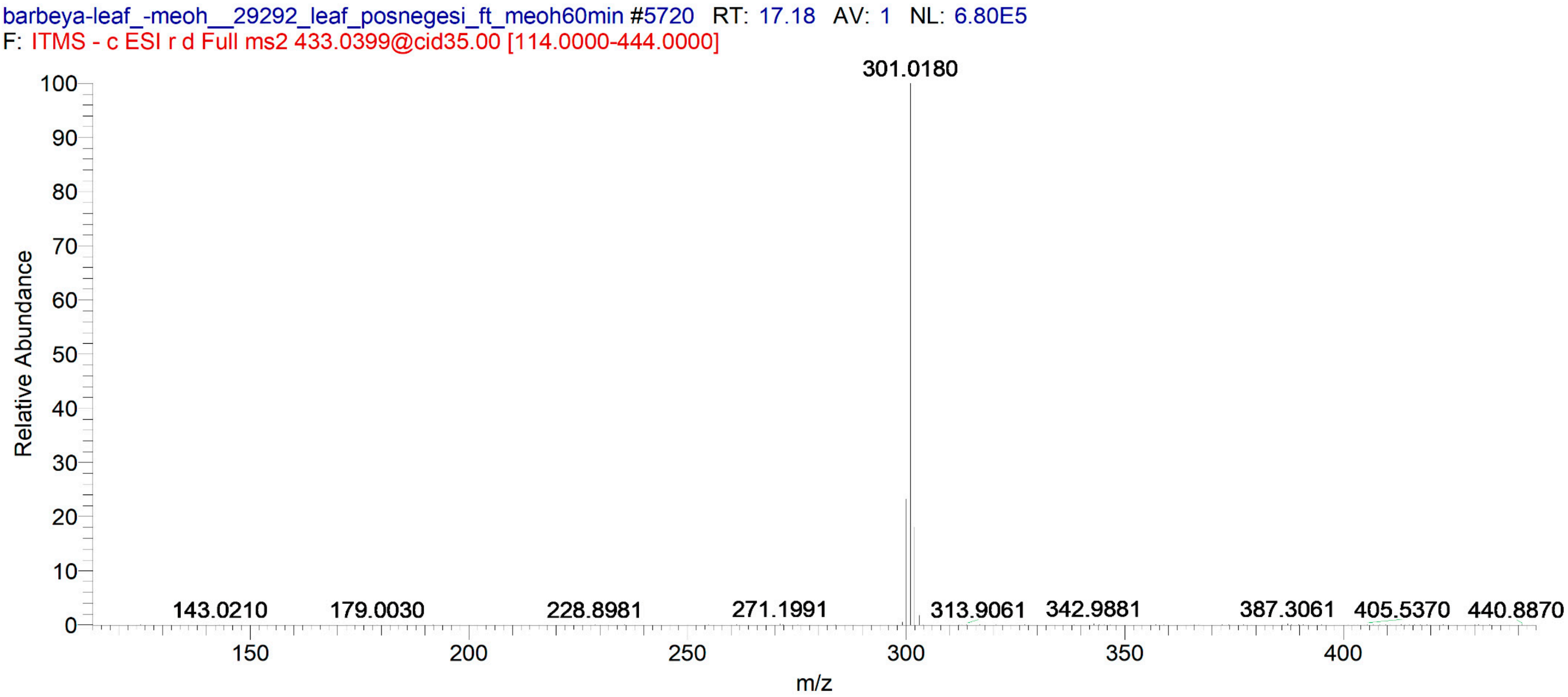

Figure S22. MS^3 spectrum of compound 14.

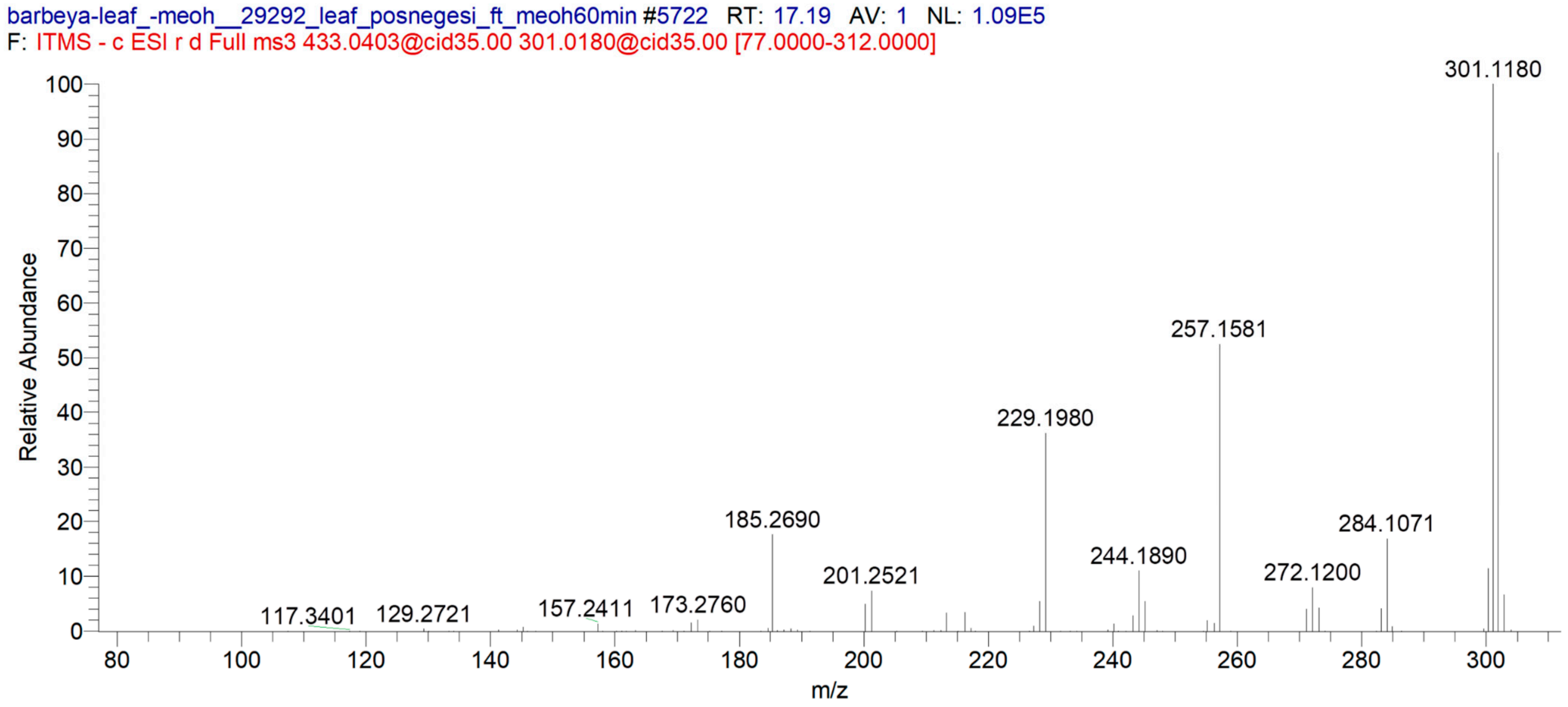

Figure S23. MS^2 spectrum of compound 15.

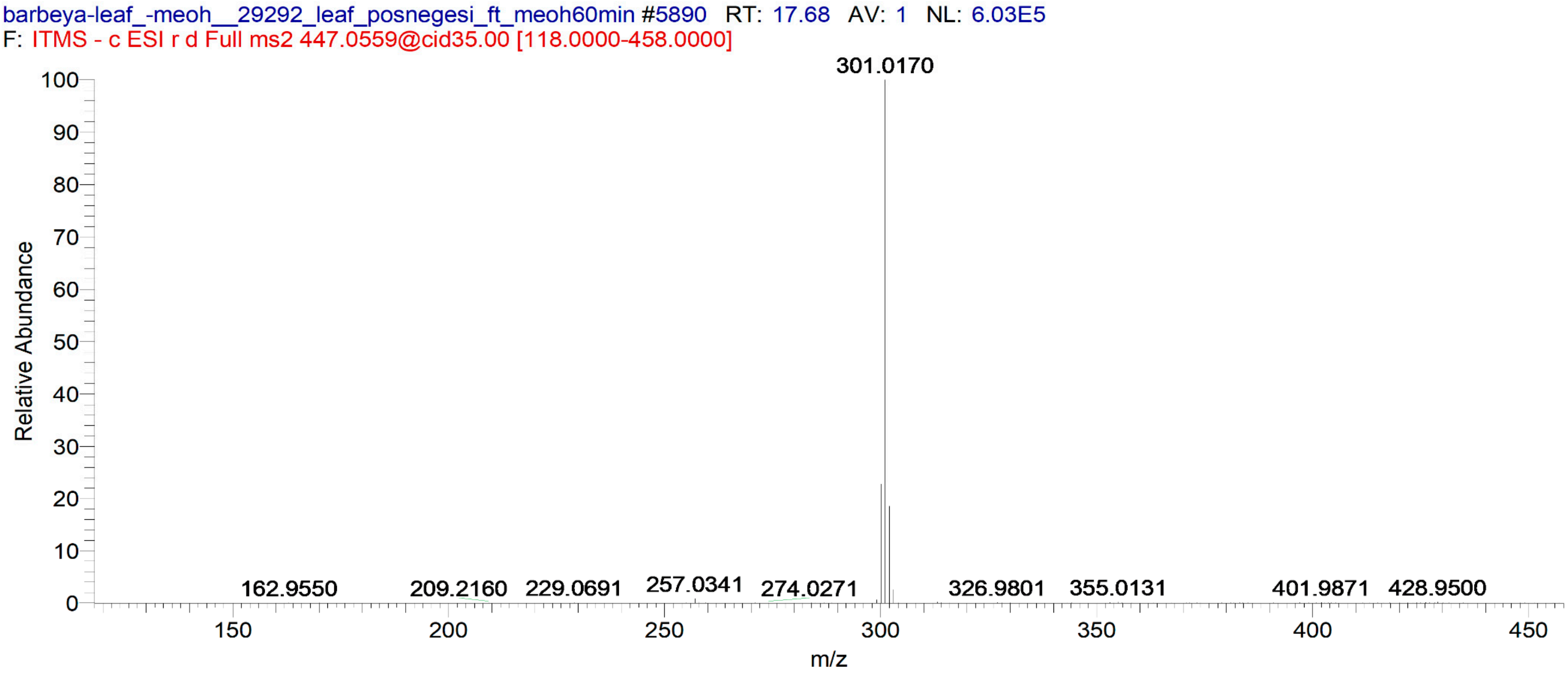

Figure S24. MS[3] spectrum of compound 15.

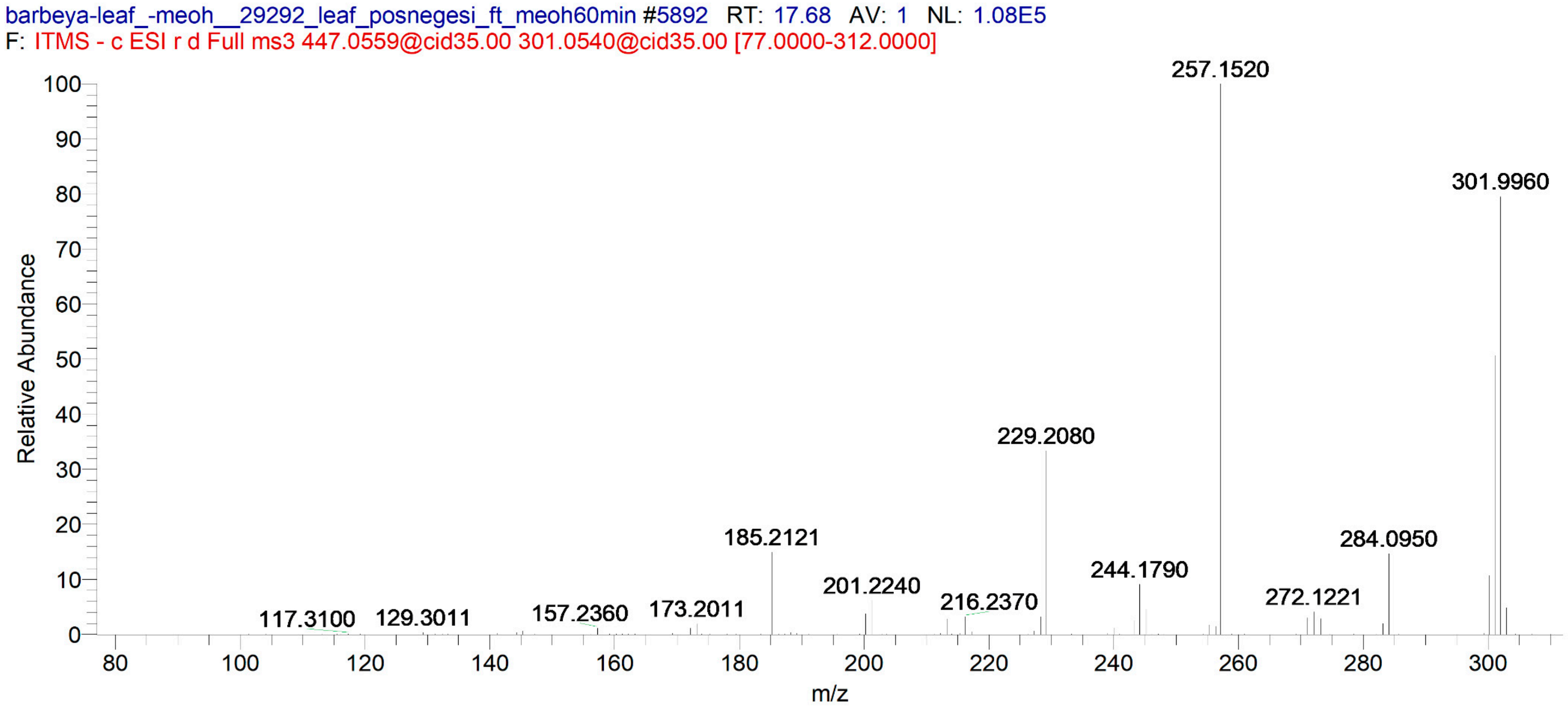

Figure S25. MS^2 spectrum of compound 16.

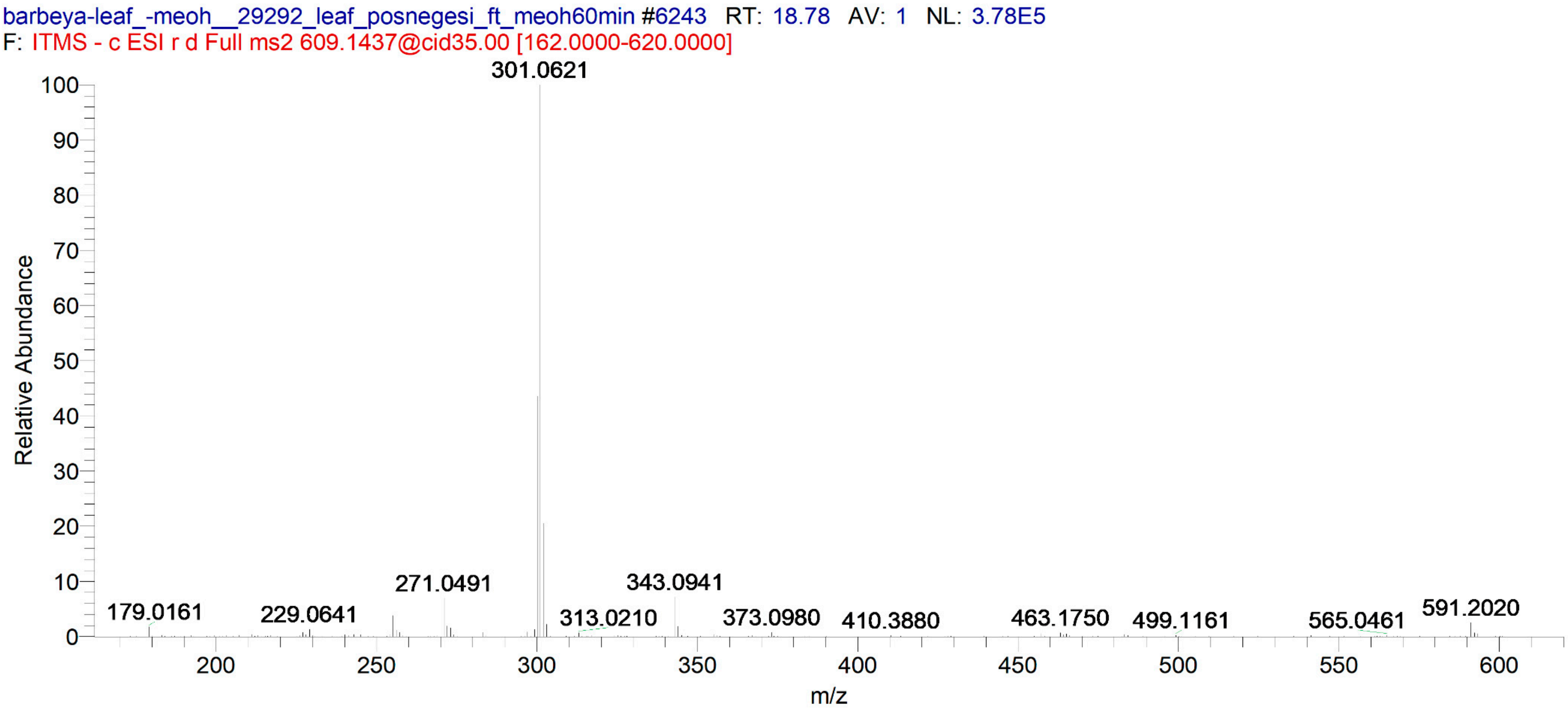

Figure S26. MS^{3}spectrum of compound 16.

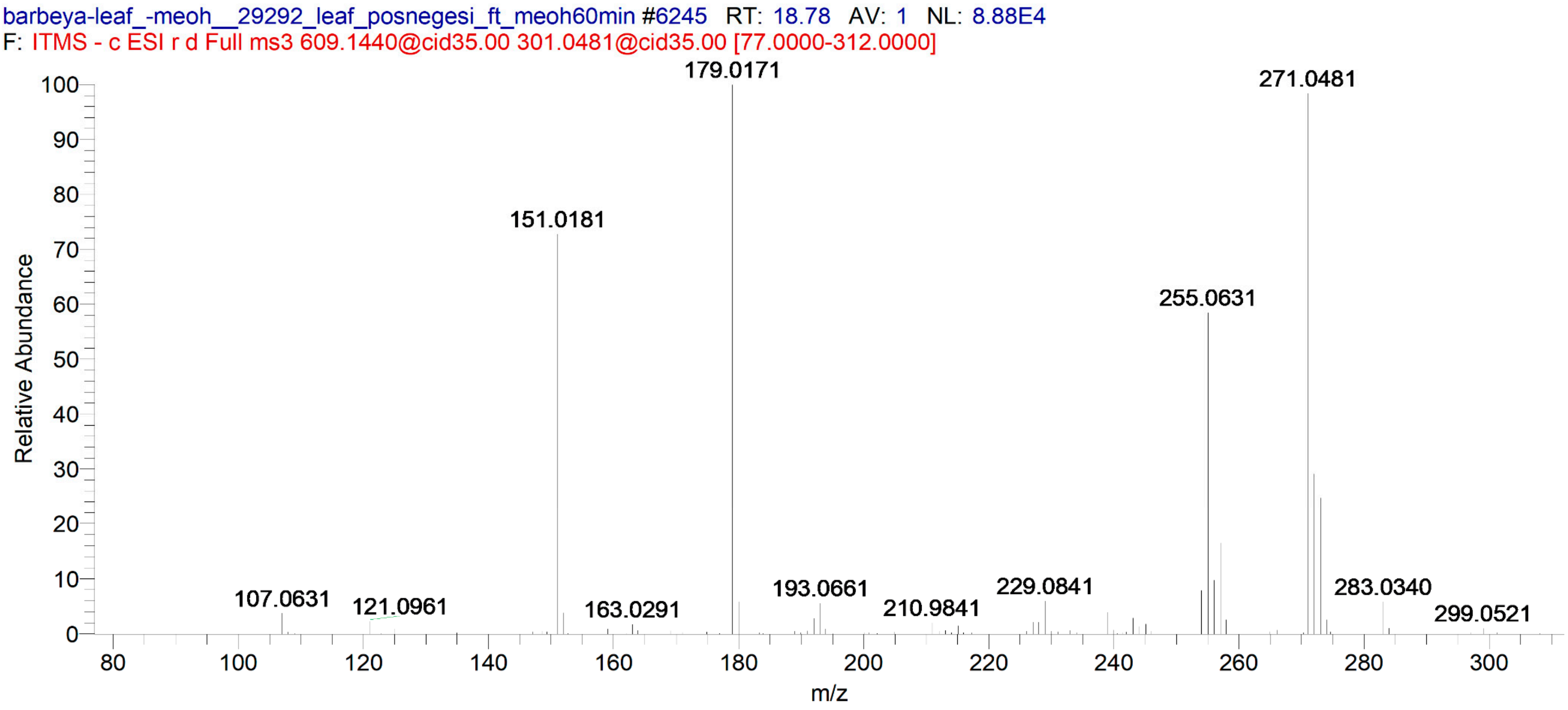

Figure S27. MS^2 spectrum of compound 17.

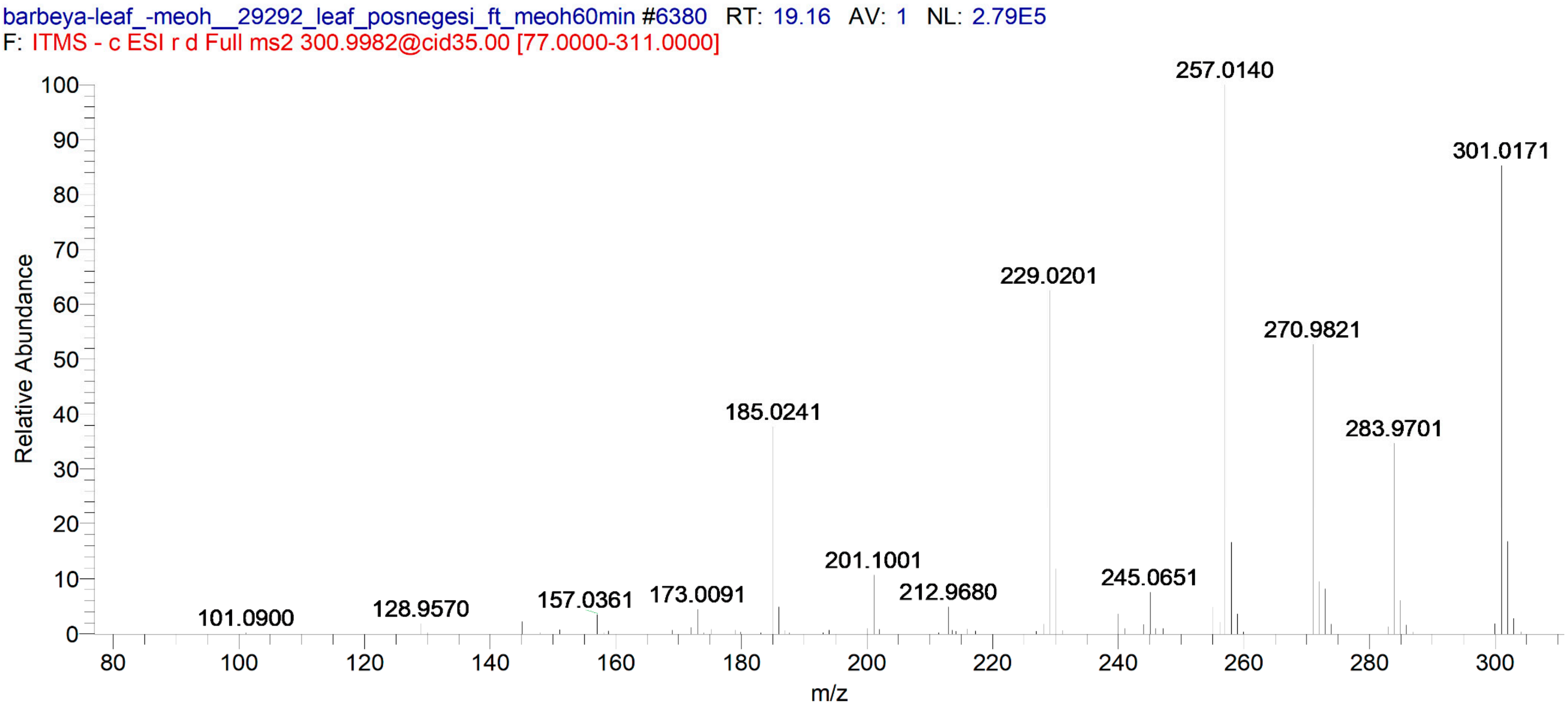

Figure S28. MS^2 spectrum of compound 18

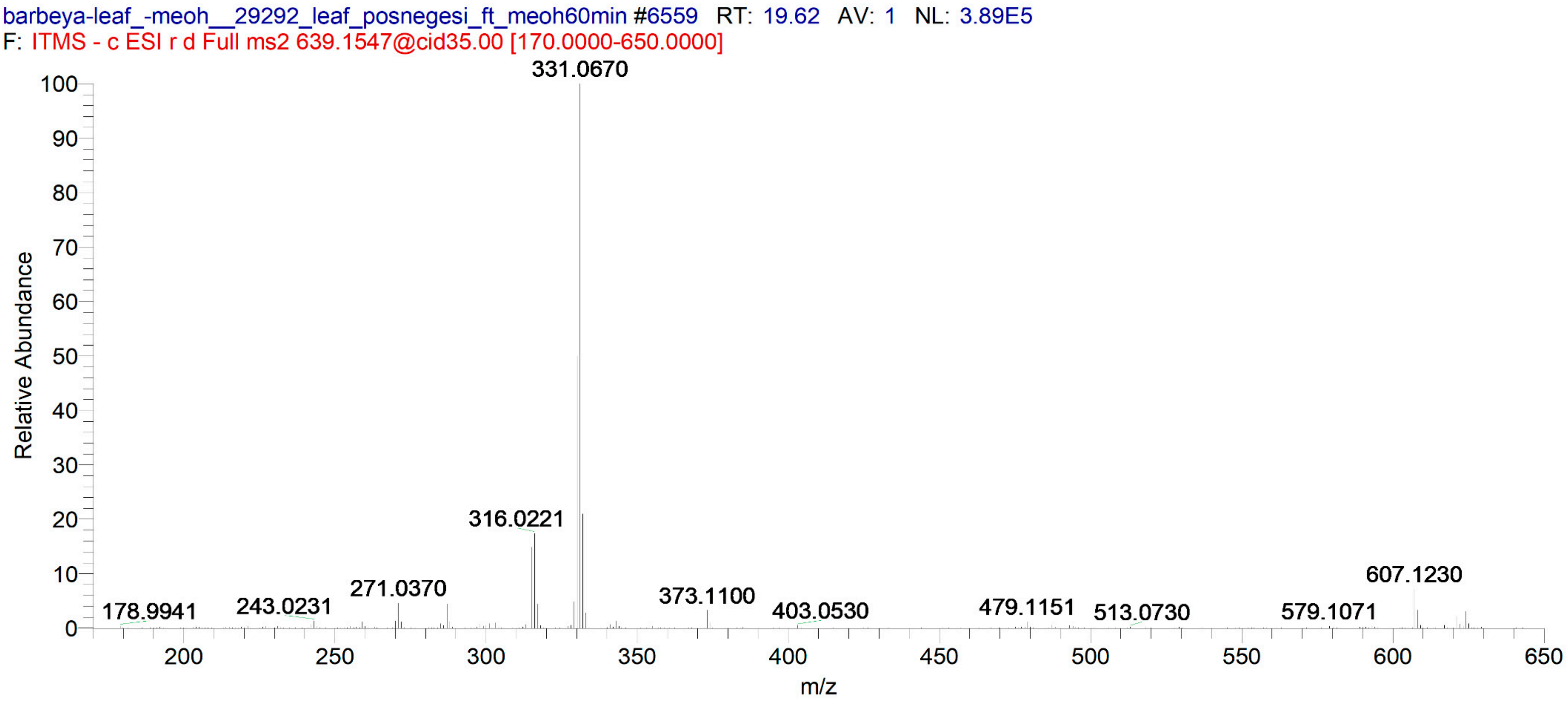

Figure S29. MS^3 spectrum of compound 18.

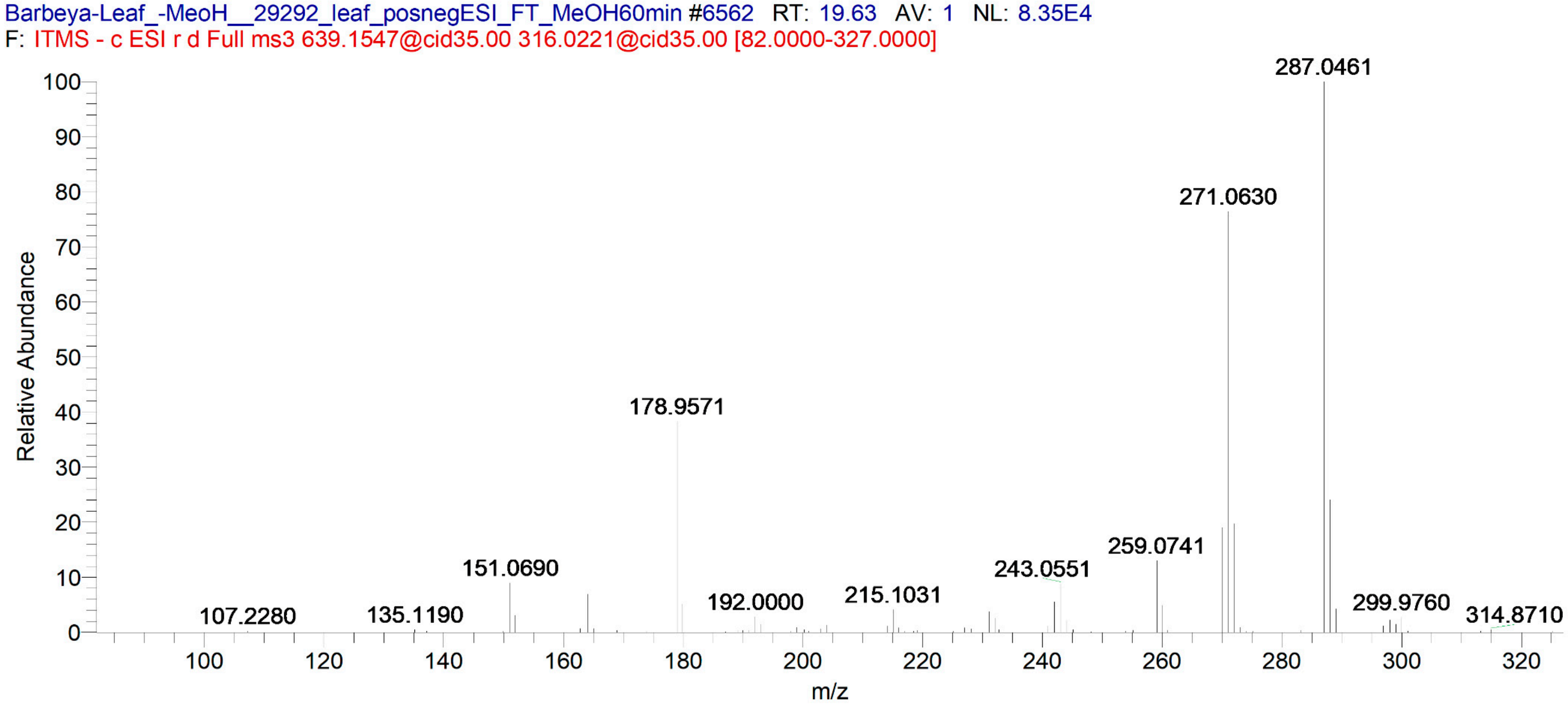

Figure S30. MS^2 spectrum of compound 19.

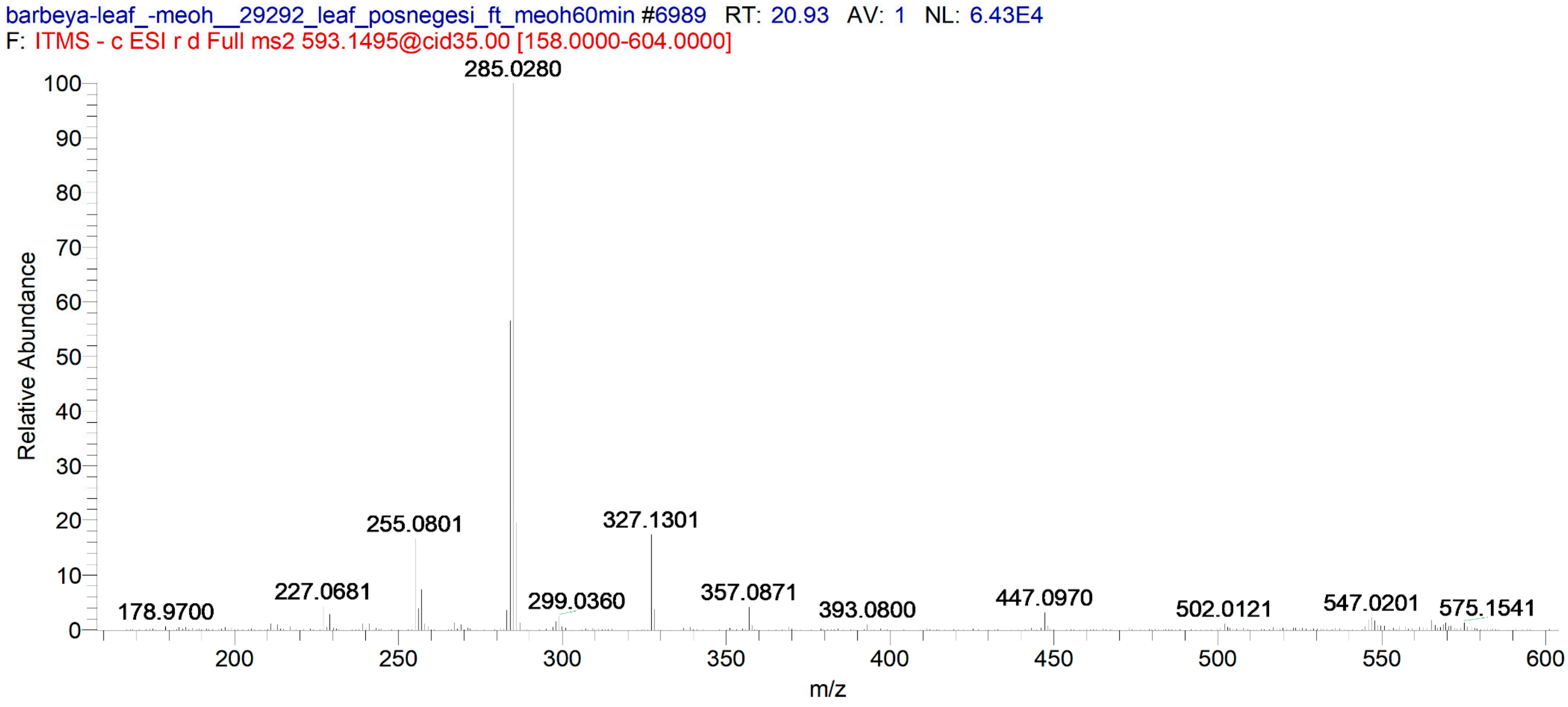

Figure S31. MS^2 spectrum of compound 20.

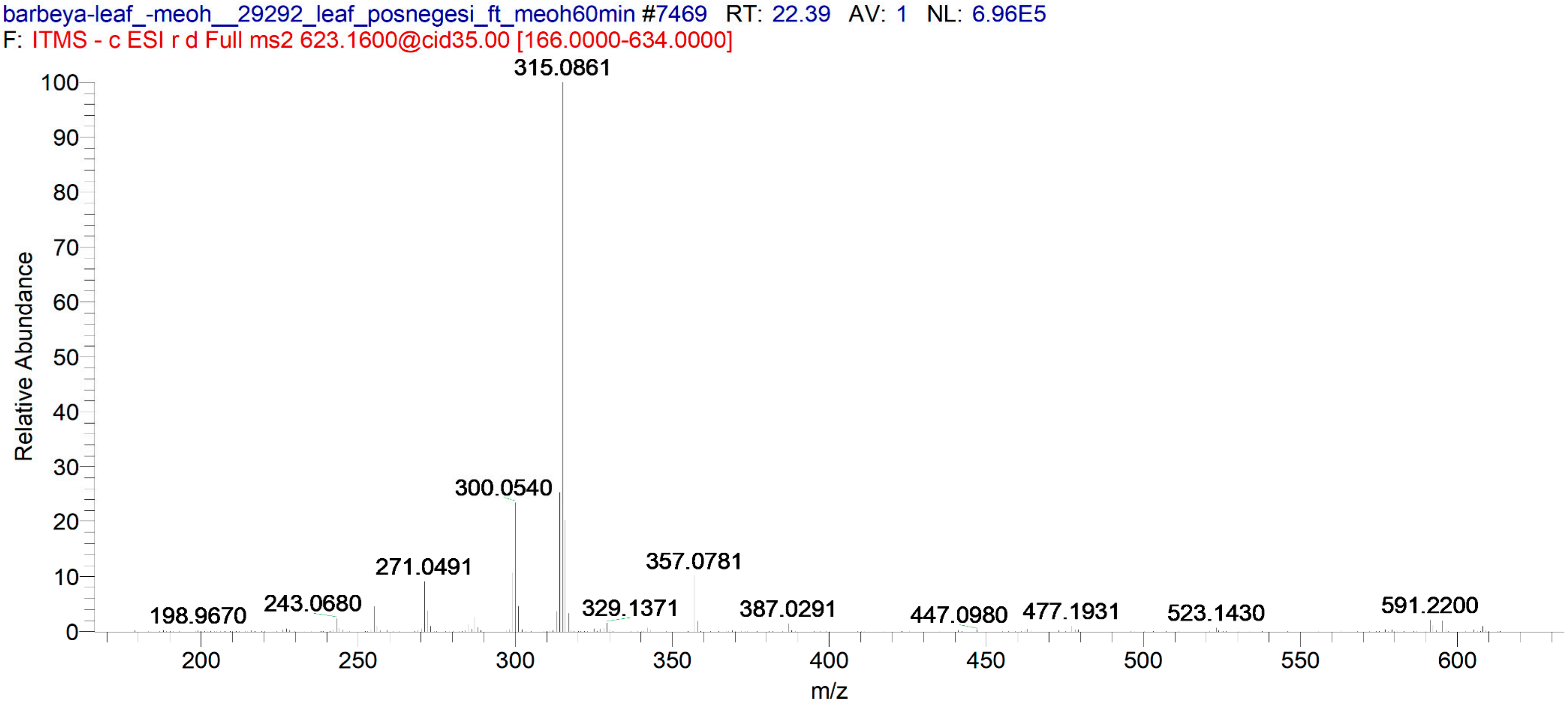

Figure S32. MS² spectrum of compound 21.

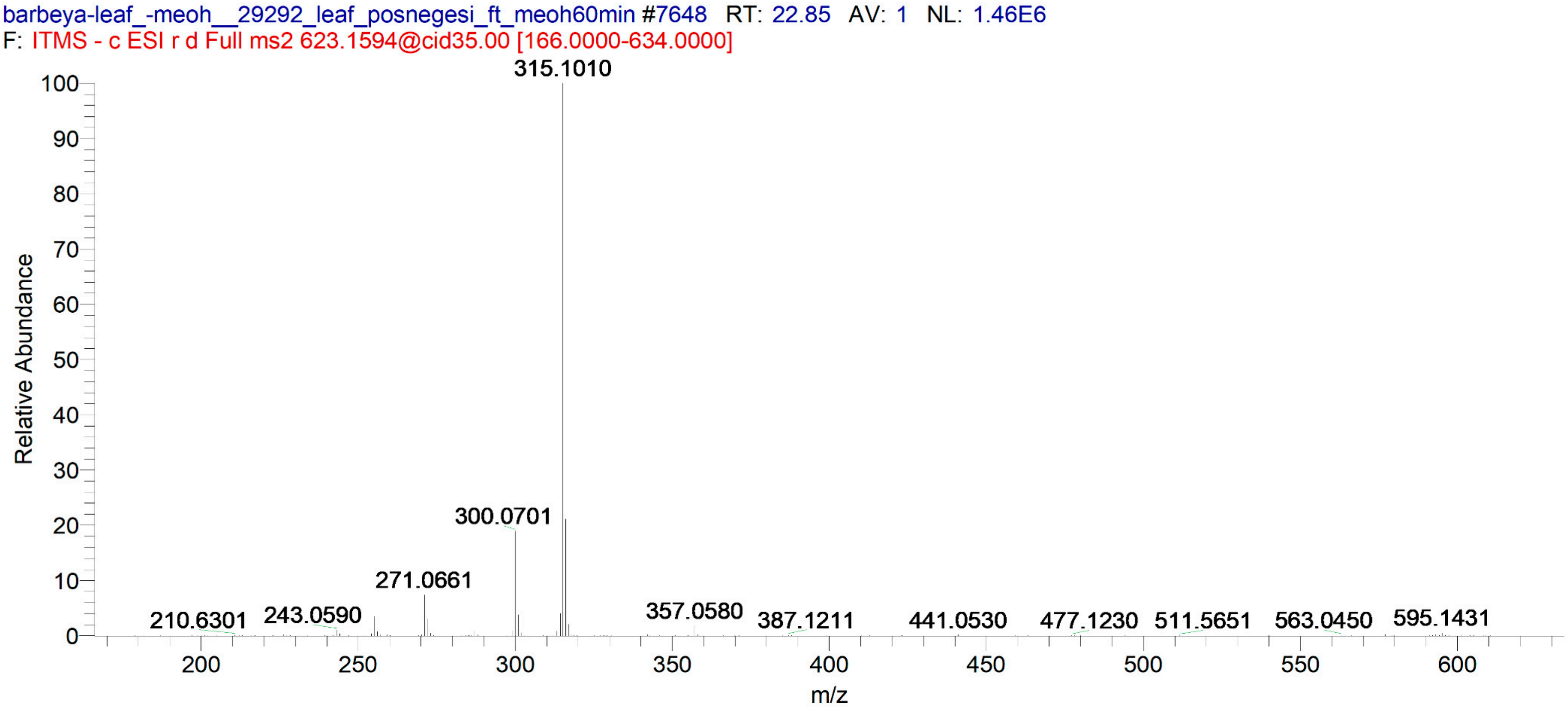

Figure S33. MS^2 spectrum of compound 22.

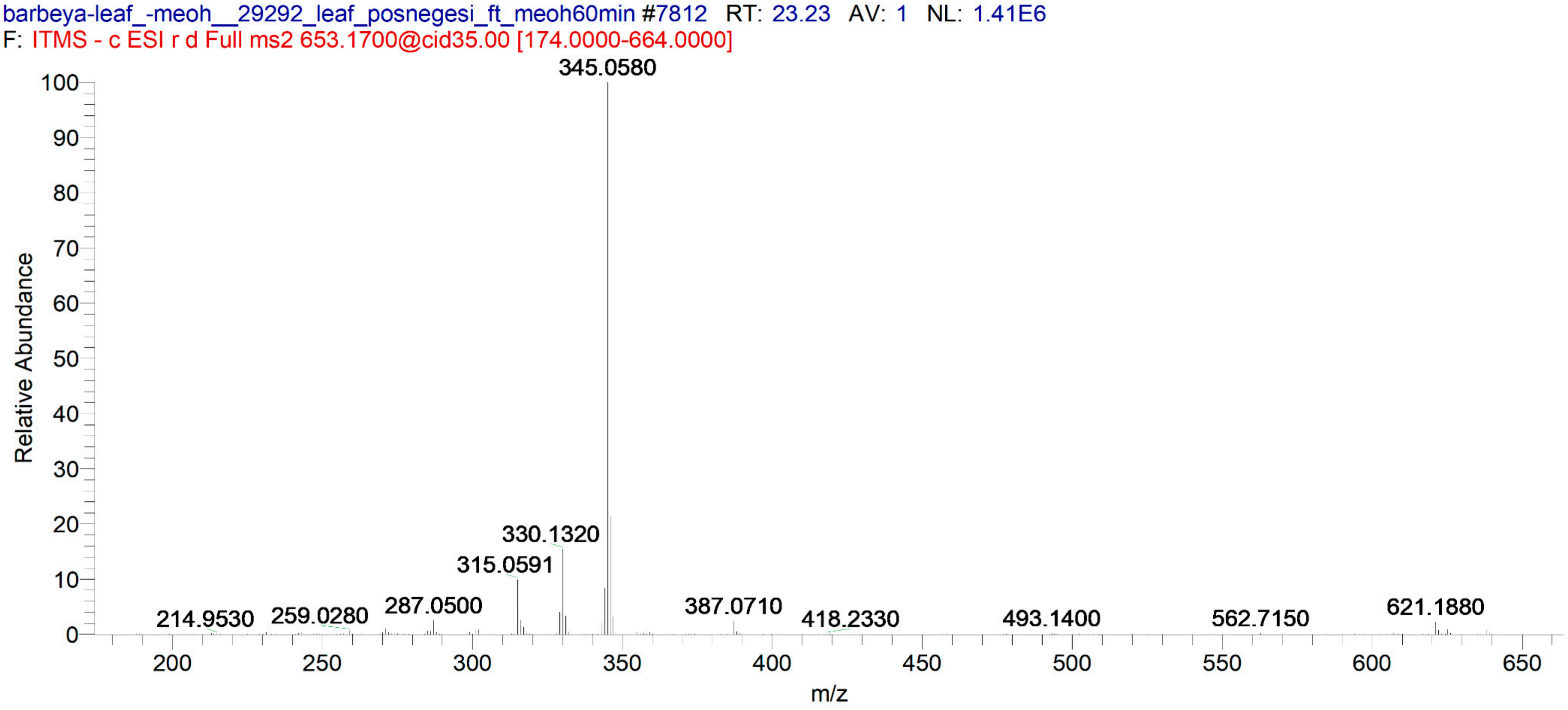

Figure S34. MS^3 spectrum of compound 22.

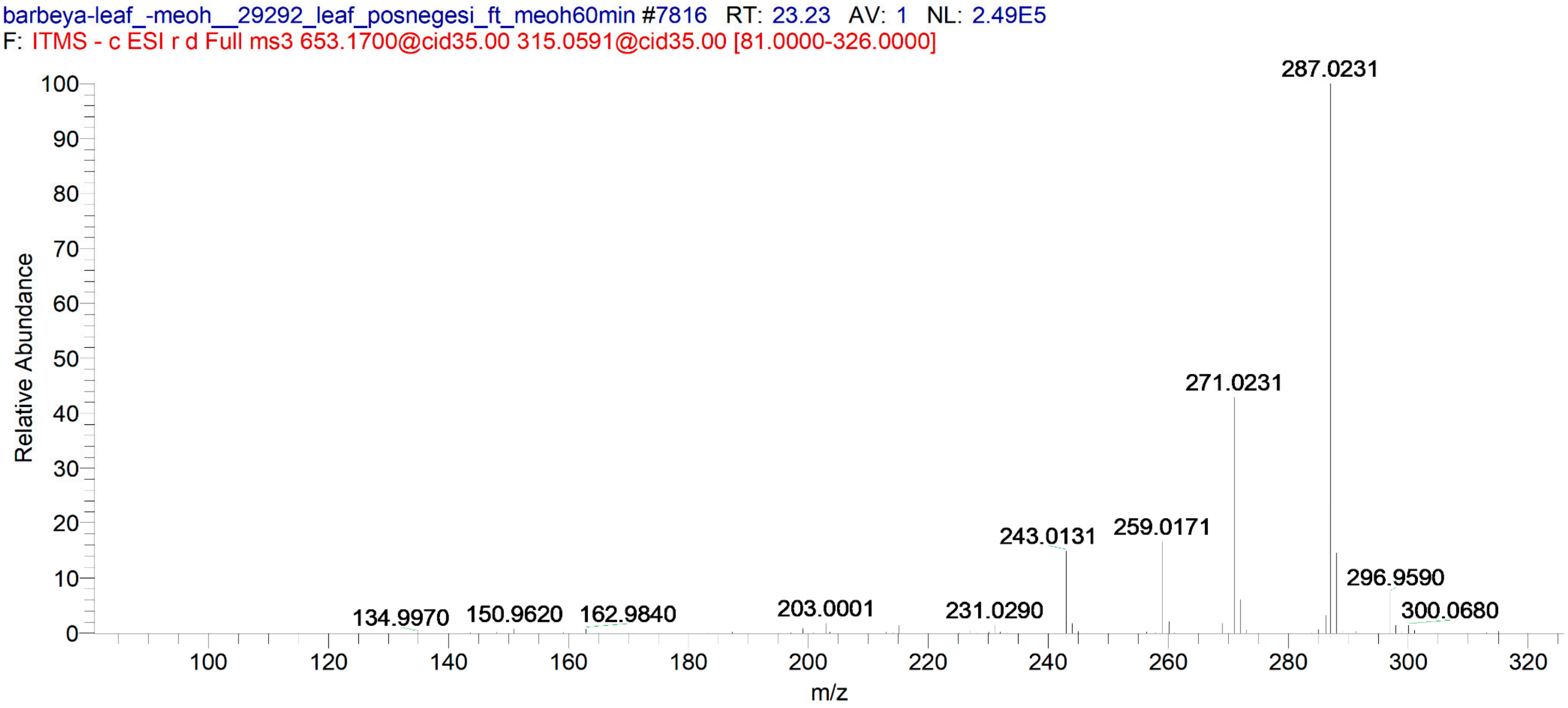

Figure S35. MS^2 spectrum of compound 23.

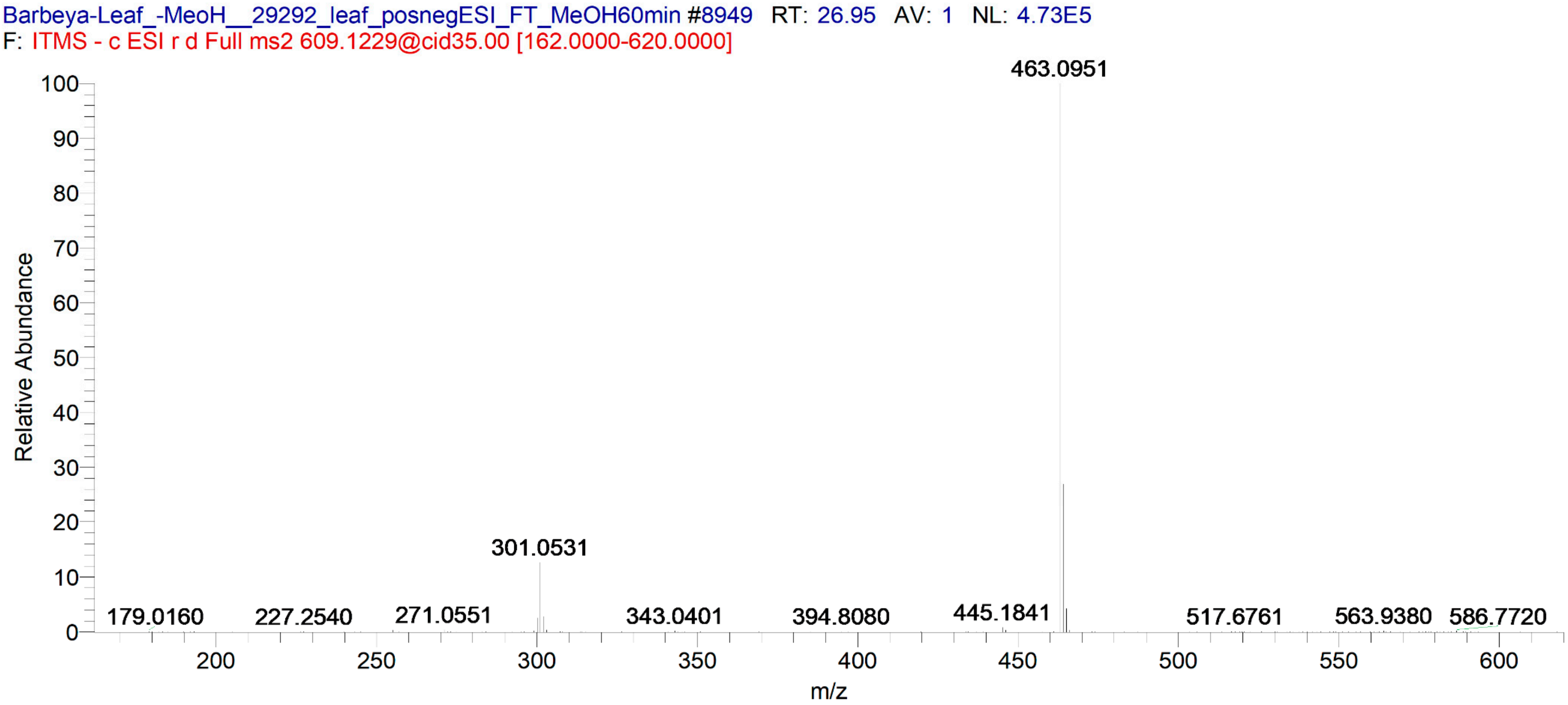

Figure S36. MS^3 spectrum of compound 23.

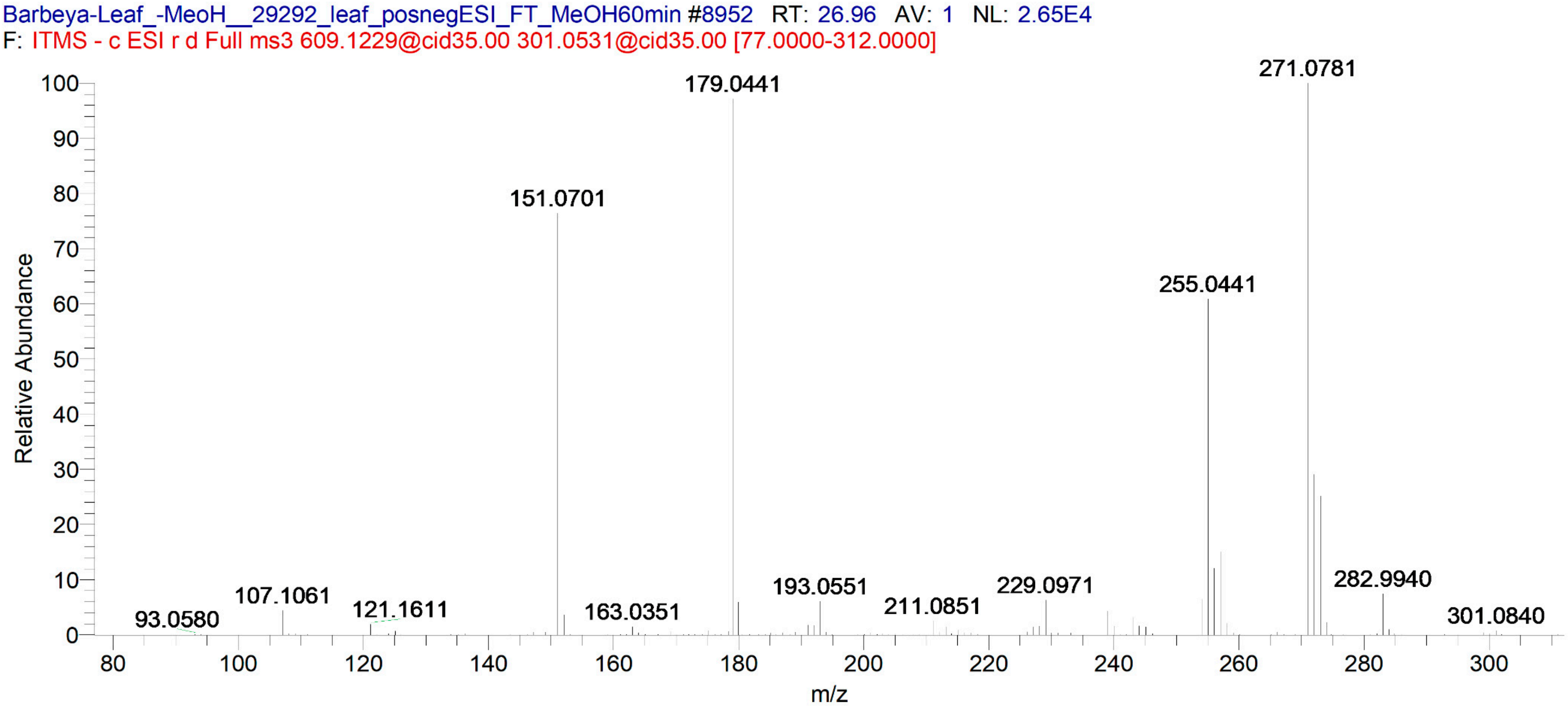

Figure S37. MS[2] spectrum of compound 24.

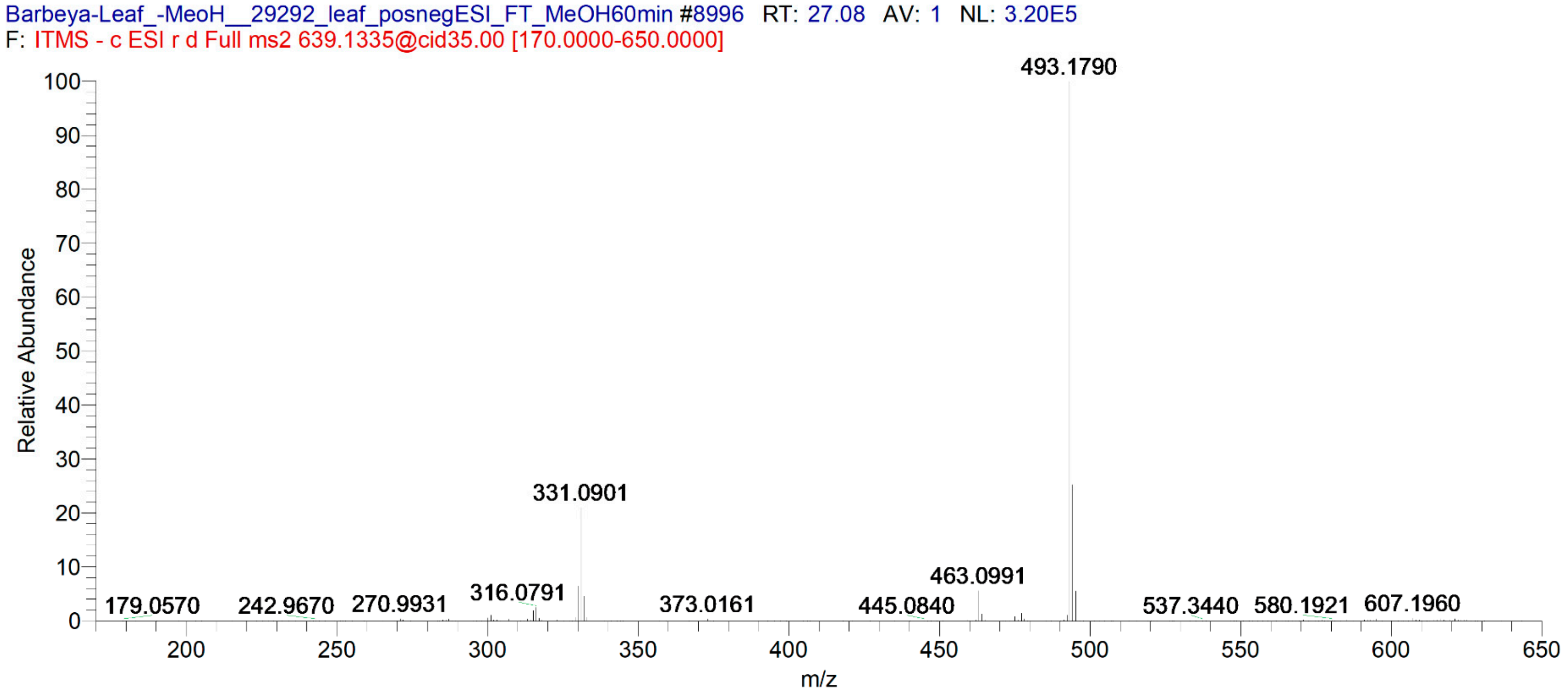

Figure S38. MS^3 spectrum of compound 24.

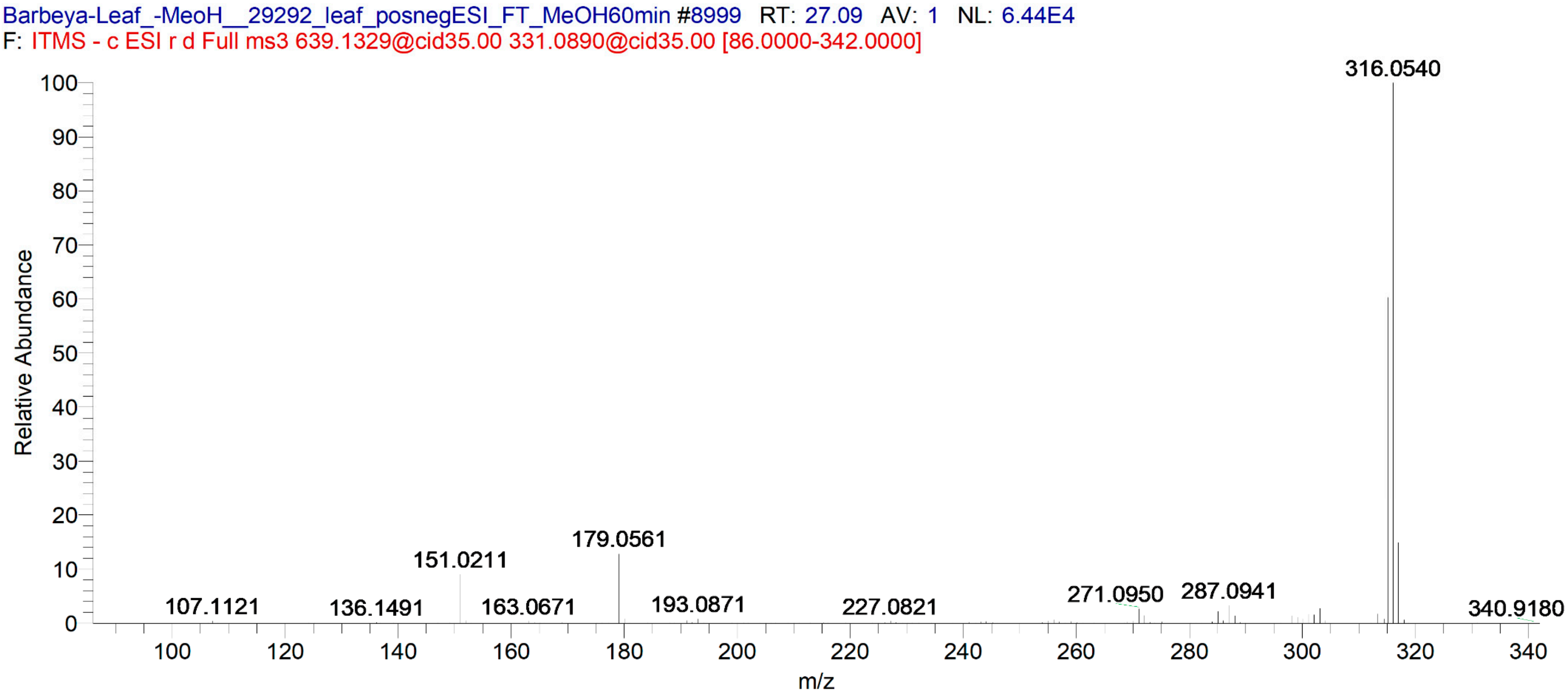

Figure S39. MS^2 spectrum of compound 25.

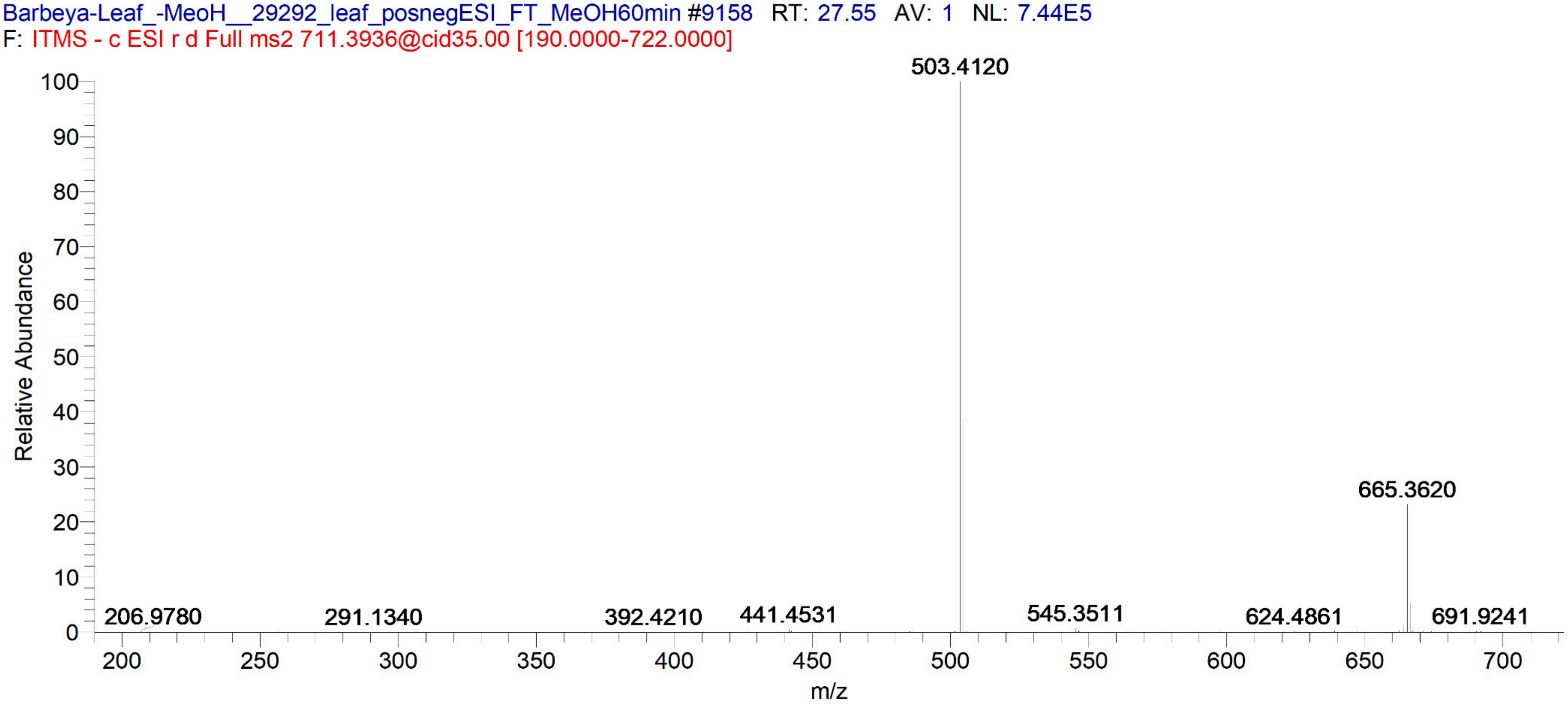

Figure S40. MS^3 spectrum of compound 25.

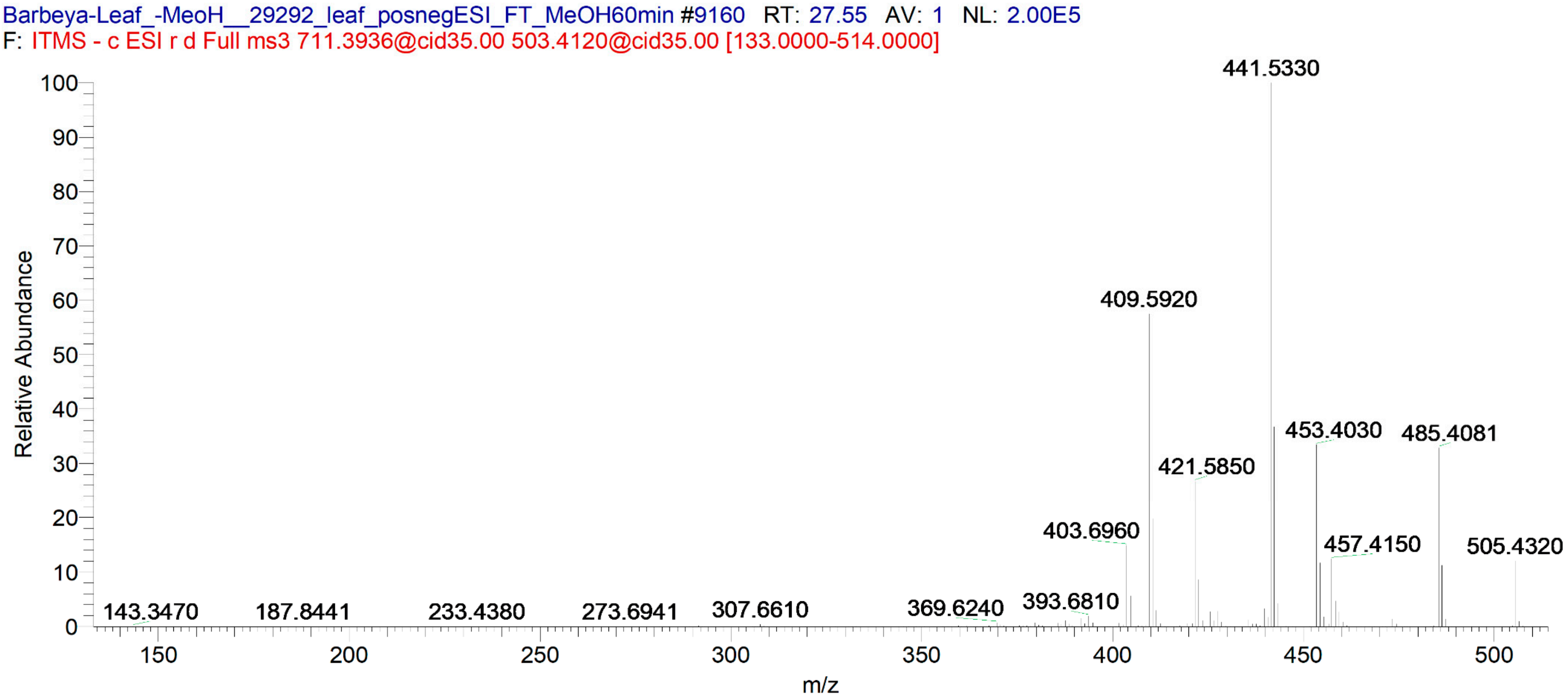

Figure S41. MS^2 spectrum of compound 26.

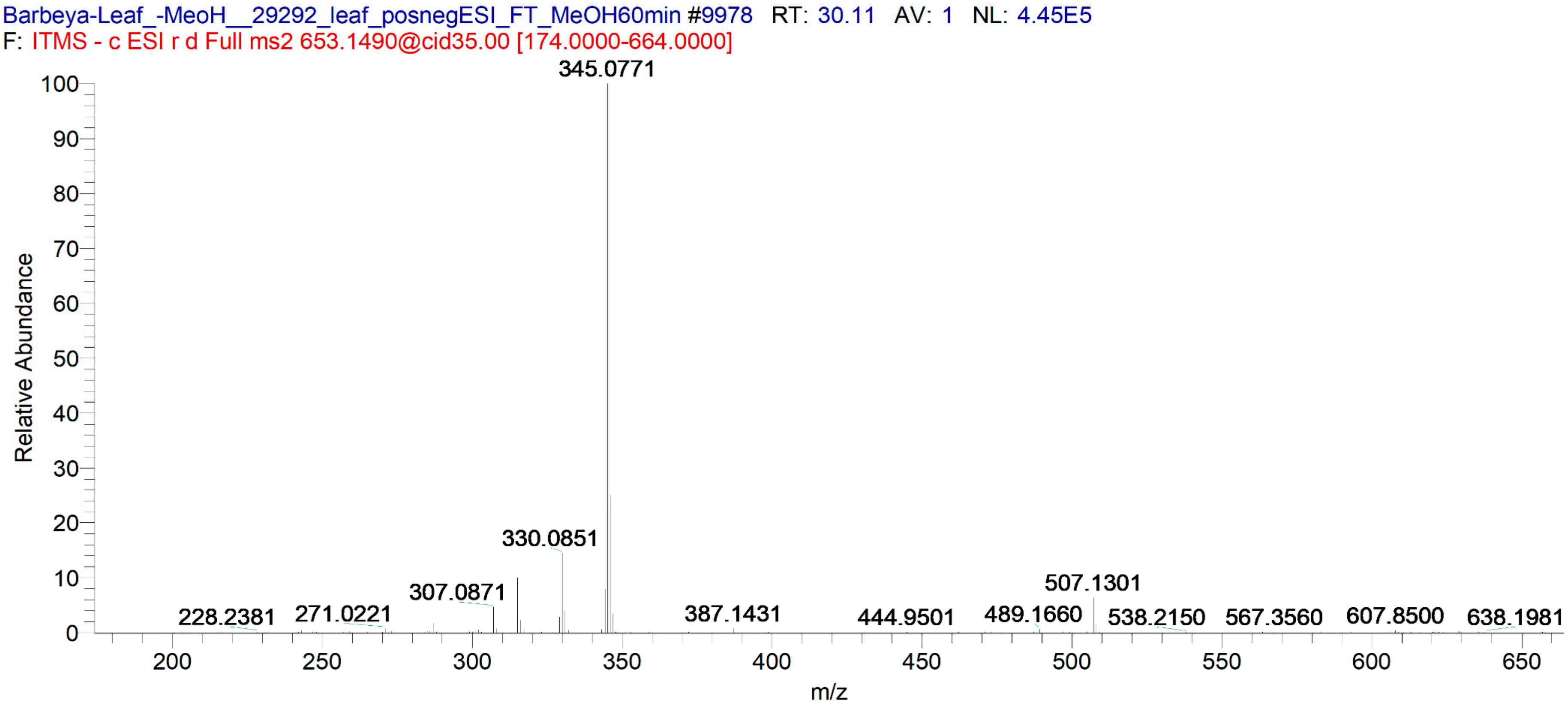

Figure S42. MS^3 spectrum of compound 26.

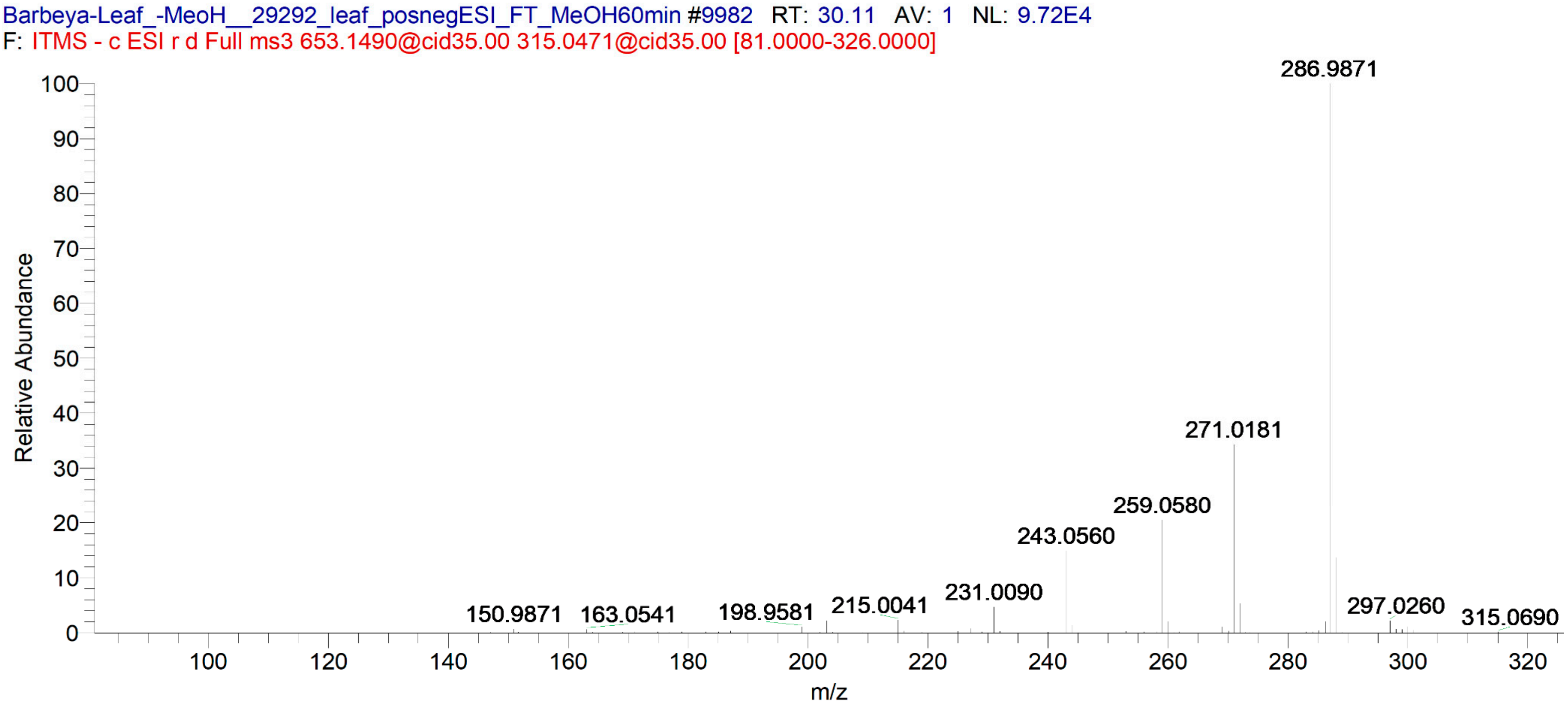

Figure S43. MS[2] spectrum of compound 27.

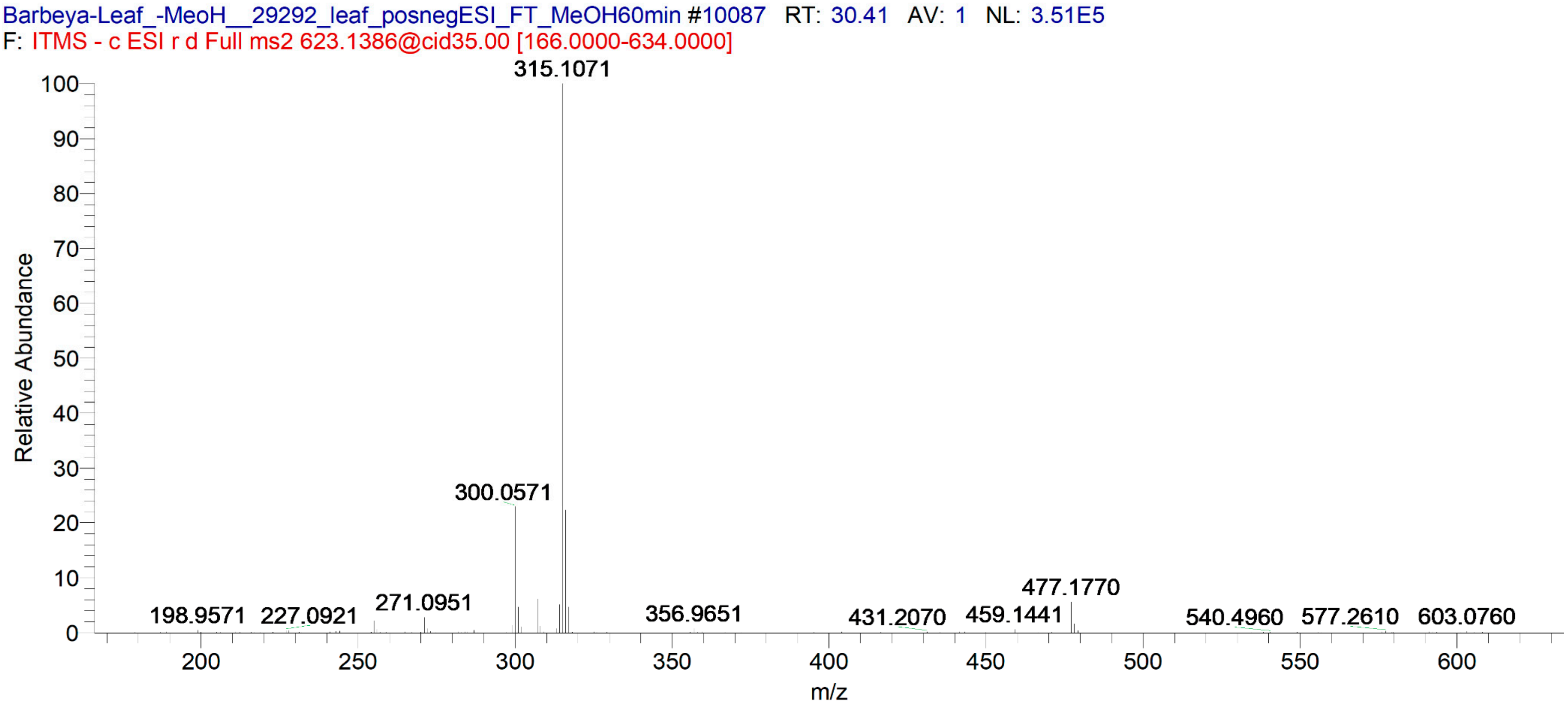

Figure S44. MS^3 spectrum of compound 27.

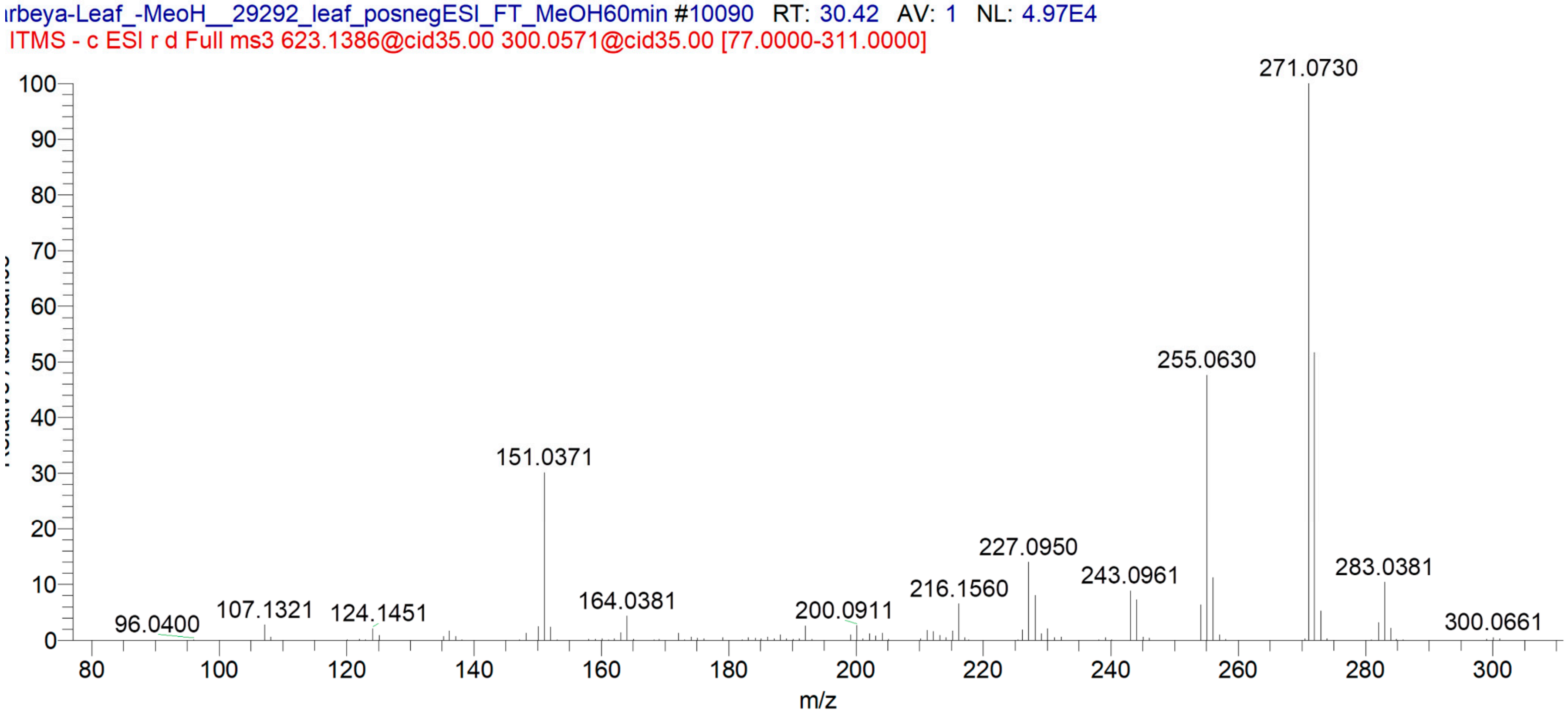

Figure S45. Dose-response curve of α-amylase inhibition by acarbose and methanol extracts obtained from *Barbeya oleoides* leaves (200 μg/ml).

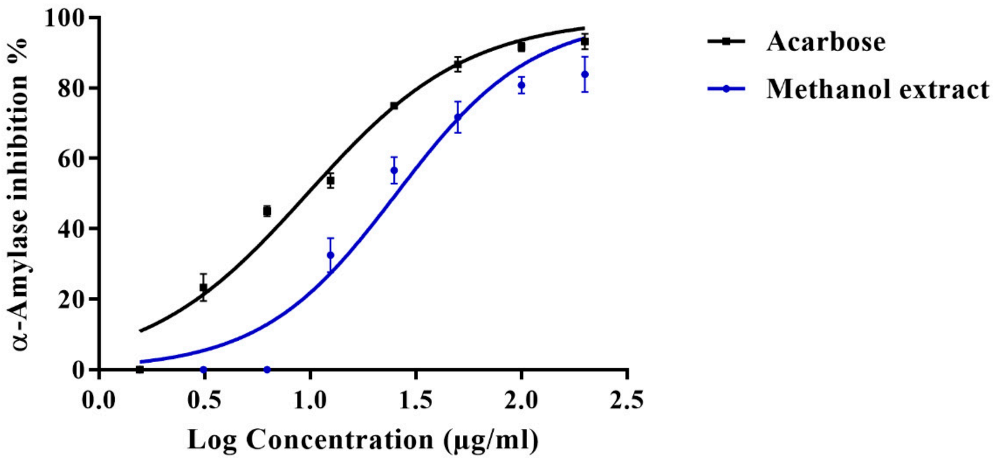

Supplement: Supplementary file 1 [file molecules-26-06229-s001.zip › molecules-1333980-supplementary.pdf]
